# Changes in retinoid metabolism and signaling associated with metabolic remodeling during fasting and in type I diabetes

**DOI:** 10.1016/j.jbc.2021.100323

**Published:** 2021-01-22

**Authors:** Alla V. Klyuyeva, Olga V. Belyaeva, Kelli R. Goggans, Wojciech Krezel, Kirill M. Popov, Natalia Y. Kedishvili

**Affiliations:** 1 Department of Biochemistry and Molecular Genetics, University of Alabama at Birmingham, Birmingham, Alabama, USA; 2 Institute of Genetics and Molecular and Cellular Biology (IGBMC) - INSERM, University of Strasbourg, Strasbourg, France

**Keywords:** vitamin A, retinol, dehydrogenase, reductase, RDH10, DHRS3, retinoic acid, liver, fasting, lipid droplets, NADPH, nicotinamide adenine dinucleotide phosphate hydrogen, RA, retinoic acid, RDH, retinol dehydrogenase, SDR, short-chain dehydrogenase/reductase

## Abstract

Liver is the central metabolic hub that coordinates carbohydrate and lipid metabolism. The bioactive derivative of vitamin A, retinoic acid (RA), was shown to regulate major metabolic genes including phosphoenolpyruvate carboxykinase, fatty acid synthase, carnitine palmitoyltransferase 1, and glucokinase among others. Expression levels of these genes undergo profound changes during adaptation to fasting or in metabolic diseases such as type 1 diabetes (T1D). However, it is unknown whether the levels of hepatic RA change during metabolic remodeling. This study investigated the dynamics of hepatic retinoid metabolism and signaling in the fed state, in fasting, and in T1D. Our results show that fed-to-fasted transition is associated with significant decrease in hepatic retinol dehydrogenase (RDH) activity, the rate-limiting step in RA biosynthesis, and downregulation of RA signaling. The decrease in RDH activity correlates with the decreased abundance and altered subcellular distribution of RDH10 while *Rdh10* transcript levels remain unchanged. In contrast to fasting, untreated T1D is associated with upregulation of RA signaling and an increase in hepatic RDH activity, which correlates with the increased abundance of RDH10 in microsomal membranes. The dynamic changes in RDH10 protein levels in the absence of changes in its transcript levels imply the existence of posttranscriptional regulation of RDH10 protein. Together, these data suggest that the downregulation of hepatic RA biosynthesis, in part *via* the decrease in RDH10, is an integral component of adaptation to fasting. In contrast, the upregulation of hepatic RA biosynthesis and signaling in T1D might contribute to metabolic inflexibility associated with this disease.

Numerous studies have demonstrated that the bioactive derivative of vitamin A, retinoic acid (RA), is essential for proper embryonic development (1, 2, 3), robust immune responses (4, 5), male fertility (6, 7), epigenetic regulation (8, 9, 10), and maintenance of circadian rhythms (11, 12, 13). Especially well established is the role of RA during embryogenesis (2, 3). The concentration of RA in developing embryos changes dynamically in a strictly defined spatiotemporal pattern to control the expression of precise subsets of genes in accord with developmental stage (14, 15, 16). However, little is known about the regulation of RA biosynthesis in adult tissues. It is generally believed that once the tissues have fully differentiated, the RA levels are maintained within a narrow range optimal for each type of cells (17, 18).

The liver is the central metabolic hub that coordinates carbohydrate and lipid metabolism. In the fed state, the liver tends to be glycolytic, glycogenic, and lipogenic (19, 20), whereas during fasting the liver switches to oxidation of fatty acids (FAs) as primary fuels and becomes gluconeogenic, glycogenolytic, and ketogenic. This transition requires significant metabolic remodeling that involves changes in expression levels of key metabolic enzymes. Importantly, expression levels of some of these enzymes, such as phosphoenolpyruvate carboxykinase (PEPCK), fatty acid synthase (FAS), carnitine palmitoyltransferase 1 (CPT1), and glucokinase (GCK), are also regulated by pharmacologically applied RA (21, 22, 23, 24, 25, 26, 27). The limited literature data available to date indicate that the transition from fasted to refed state is associated with changes in vitamin A metabolism in the liver (28), suggesting that vitamin A metabolism and energy metabolism are interconnected. However, very little is currently known about the regulation of RA biosynthesis and signaling in liver. This study was undertaken in order to determine whether the rate of RA biosynthesis and RA signaling in the liver change under conditions associated with significant metabolic remodeling such as fed-to-fasted transition or metabolic diseases such as type 1 diabetes (T1D).

## Results

### Hepatic metabolic remodeling during fed-to-fasted transition

**Figure 1:**
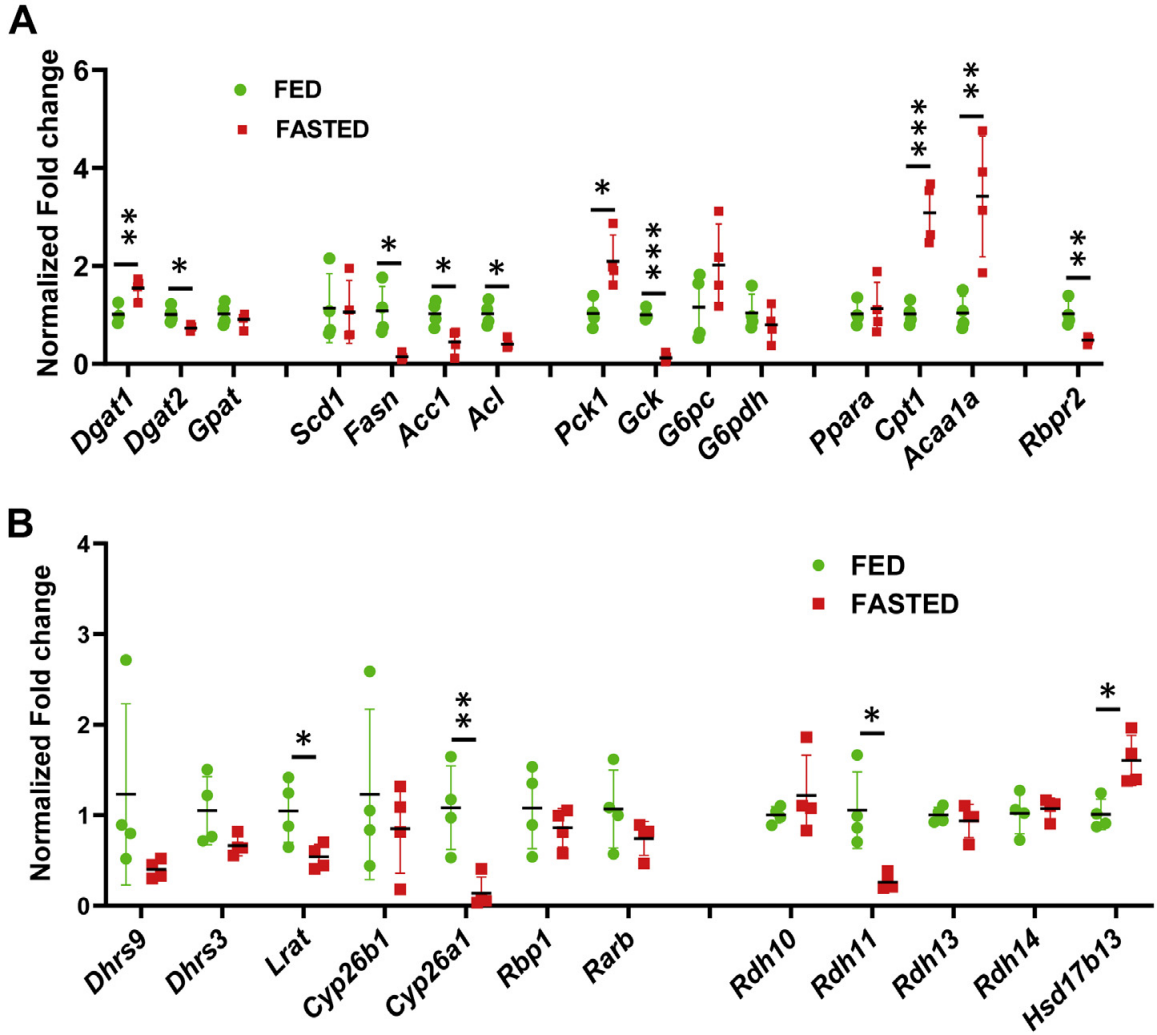
**Hepatic gene expression levels in fed *versus* 16 h-fasted mice.***A*, expression of lipid and carbohydrate metabolic genes. *B*, expression of RA metabolic and RA target genes. qPCR analysis was performed using mRNA isolated from the livers of 3-month-old fed or 16 h-fasted male mice (n = 4 for each group). ∗*p* < 0.05; ∗∗*p* < 0.01; ∗∗∗*p* < 0.001. *Error bars*, S.D.

To characterize the dynamics of RA signaling during the fed-to-fasted transition, we chose two time points: 16 h and 24 h after beginning of fasting. As would be expected from the earlier studies (29), the livers of fasted mice underwent a major metabolic remodeling. QPCR analysis showed that liver samples obtained from 16 h-fasted mice exhibited clear signs of upregulated FA oxidation, as evidenced by the increase in expression levels of carnitine palmitoyltransferase 1 (*Cpt1*); downregulated ability to uptake blood glucose (decrease in glucokinase (*Gck*)); upregulated gluconeogenesis (increase in phosphoenolpyruvate carboxykinase (*Pck1*); and downregulated lipogenesis (decrease in FA synthase (*Fasn*), ATP citrate lyase (*Acl*), and acetyl-CoA carboxylase 1 (*Acc1*)) (Fig. 1*A*). Gene expression levels were normalized to geometric mean of four housekeeping genes’ expression, which was relatively stable between the fed and fasted groups (Fig. S1). Similar changes in the expression of metabolic genes were observed in the livers of mice fasted for 24 h (Fig. S2*A*).

To determine whether metabolic adaptation to fasting affected the retinoid metabolism and RA signaling, we examined the expression levels of retinoid metabolic genes and genes regulated by RA. This analysis revealed that liver samples from 16 h-fasted mice displayed a significant downregulation in the expression of retinoid metabolic genes: *Cyp26a1*, *Lrat*, and *Rdh11* (Fig. 1*B*). Particularly remarkable was an approximately tenfold decrease in the expression of *Cyp26a1*. In the liver, this low-affinity but high-activity enzyme is believed to be primarily responsible for the disposal of excessive RA (30, 31). Furthermore, it was established that its expression is highly sensitive to RA levels (32, 33). The liver samples from 24 h-fasted mice displayed a similar pattern, with statistically significant decreases observed in the expression of *Rbp1* and *Dhrs9* in addition to *Cyp26a1* and *Rdh11* (Fig. S2*B*). Therefore, the dramatic decrease in expression of *Cyp26a1* along with downregulation in expression of *Lrat*, which is also an RA-sensitive gene (34), and decreases in *Rbp1* and *Rdh11* expression suggest that the extensive metabolic remodeling that occurs during the fed-to-fasted transition might include adaptive changes in retinoid metabolism and signaling.

### Expression and subcellular localization of hepatic RDH10 and DHRS3 in fed and fasted states

**Figure 2:**
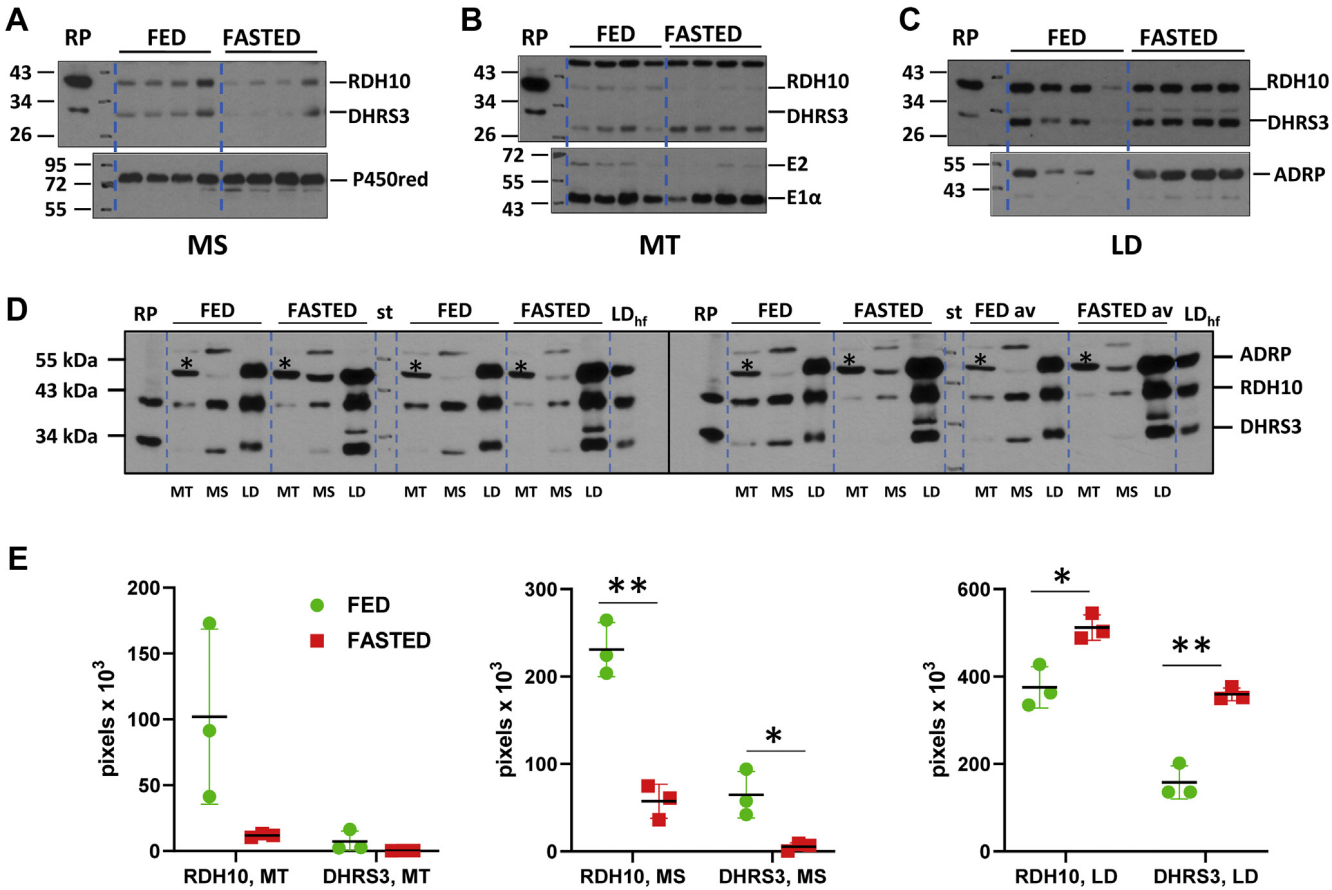
**Protein levels of RDH10 and DHRS3 in hepatic membrane fractions from fed *versus* fasted mice.***A*–*C*, side-by-side western blot analysis of hepatic subcellular fractions from mice fasted for 16 h. Subcellular fractions were isolated from four individual 3-month-old male mice and loaded as follows: 40 μg of hepatic microsomes (*A*); 40 μg of mitochondria (*B*); and lipid droplets loaded by wet weight (10 μl out of 200 μl) (*C*). The blot was incubated with a mixture of RDH10 and DHRS3 antibodies diluted 1:3000 each. After developing the blots, they were incubated with the corresponding markers of each subcellular fraction: P450 reductase (P450red), antibodies diluted 1:4000; pyruvate dehydrogenase complex subunits E2 and E1α, antibodies diluted 1:5000; and ADRP (perilipin 2), antibodies diluted 1:3000. *D*, western blot analysis of RDH10 and DHRS3 protein content in mitochondria (MT), microsomes (MS), and lipid droplets (LD) isolated from 3-month-old male mice (n = 3) fed or fasted for 24 h. Each membrane fraction was resuspended in 200 μl, the MS and MT membrane fractions (but not LD fraction) were diluted fivefold and 10 μl of each fraction was loaded onto two separate gels. The last sample set represents the average of fractions from individual mice mixed together (FED av and FASTED av). A representative Ponceau-stained blots showing protein amount in each lane are depicted in Fig. S5*A*. *E*, quantification of RDH10 and DHRS3 proteins in hepatic membrane fractions from fed and 24 h-fasted mice. The blots were scanned using UN-SCAN-IT and the data are presented as total pixel density. ∗*p* < 0.05; ∗∗*p* < 0.01. *Error bars*, S.D. Abbreviations are: LD_hf_, lipid droplet fraction from the livers of mice on high-fat diet; RP, recombinant untagged human RDH10 and DHRS3 proteins overexpressed in HEK293 cells. Positions of molecular mass standards are indicated by numbers; *asterisks* (∗) denote a nonspecific protein band recognized by RDH10 antibodies in mitochondrial membranes.

Cellular RA is synthesized in a two-step process [reviewed in ref. (35). The first step catalyzed by microsomal NAD^+^-dependent retinol dehydrogenase(s) (RDH) converts retinol (ROL) to retinaldehyde (RAL). The second step catalyzed by cytosolic retinaldehyde dehydrogenase(s) converts RAL to RA. Evidence parented by Napoli’s laboratory strongly suggests that the first step, catalyzed by one of the microsomal RDH(s), is the rate-limiting step for the overall process (36). Within the past decade, RDH10 emerged as a major retinol dehydrogenase responsible for the biosynthesis of RA during embryogenesis (37, 38, 39, 40). Furthermore, recently, Yang and colleagues showed that RDH10 is also a major dehydrogenase contributing to biosynthesis of RA in adult mouse liver (41). Therefore, in order to evaluate the contribution of RDH10 to biosynthesis of hepatic RA in the fed *versus* fasted state, we analyzed the cellular abundance and subcellular distribution of RDH10 using western blot analysis. Antibodies used for RDH10 detection were validated using samples of microsomal membranes isolated from RDH10 gene knockout and heterozygous mouse embryos (Fig. S3). In agreement with the results published by Yang and colleagues (41), we found that RDH10 protein was readily detectable in samples isolated from adult livers (Fig. 2). In the fed state RDH10 protein was present in heavy-, intermediate-, and low-density membrane fractions corresponding to mitochondria (MT), microsomes (MS), and lipid droplets (LD) (Fig. 2, *A*–*C*). The purity of subcellular fractions was tested using antibodies against: cytochrome P450 reductase as a marker of MS; pyruvate dehydrogenase complex subunits E2 and E1α for mitochondria; catalase as a marker of MT and peroxisomes; and perilipin 2 (ADRP) as a marker of LDs (Fig. 2 and Fig. S4). A nonspecific band present at the level of perilipin 2 in MS and MT and marked by asterisk appeared after incubation with RDH10 antibodies and does not represent perilipin 2 (Fig. S4).

Surprisingly, western blot analysis showed that 16 h fasting was associated with significant changes in the abundance of RDH10 protein. As illustrated in Figure 2, *A*–*C*, in the livers of fasted mice, RDH10 protein was decreased in MT and MS fractions, but it was increased in LD fractions compared with the livers from fed mice. It is well established that hepatic LDs increase in size and abundance in response to fasting to reserve lipids that are mobilized from the adipose tissue to the liver as a mean to provide energy for vital cellular functions (42, 43). Since the amount of LD membranes appeared to increase relative to the amount of MS and MT, we sought to assess the abundance of RDH10 protein in each membrane fraction by comparing the RDH10 levels in equal parts of LDs, MS, and MT membranes isolated from the fed *versus* fasted livers. To achieve this, we fractionated liver samples and resuspended the isolated MT, MS, and LDs membranes in 200 μl each. Protein-rich MS and MT fractions were diluted fivefold, to avoid gel overloading, whereas protein-poor LDs were used as is. Ten microliters of diluted MT and MS membranes and undiluted LDs were separated by SDS-PAGE. The total amount of loaded protein for each fraction is shown in Fig. S5*A*. For an additional control, we tested whether the purity of fractions was affected by fasting. Immunoblotting of fractions with antibodies against catalase, cytochrome P450 reductase, and perilipin 2 showed that the purity of fractions did not appear to be affected by fasting (Fig. S6). RDH10 protein levels detected by western blotting were quantified as described in the legend to Figure 2 and normalized per gram of liver wet weight. Figure 2*E* shows the observed changes in the content of RDH10 protein in MT, MS, and LD fractions after fasting. In agreement with the data presented in Figure 2, *A*–*C*, this analysis showed that fasting was associated with a decrease of RDH10 protein amount in MS and MT and an increase of RDH10 in LDs.

Interestingly, when corrected for the 5x dilution factor for MS and MT, the total amount of RDH10 protein was the highest in MS fraction in the livers from fed mice followed by LDs (Fig. S7*A*). However, after fasting, this order was reversed, with the total amount of RDH10 protein becoming greater in LDs than in MS (Fig. S7*A*). MT accounted for the lowest amount of RDH10 in fed or fasted states. The combined amount of hepatic RDH10 in all fractions decreased in fasted livers (Fig. S7*B*). Of note, considering that LD fractions contained the least amount of protein (Fig. S5*A*), LD fractions appeared to be highly enriched with respect to RDH10 in comparison with MT and MS fractions (Fig. 2*D* and Fig. S7*A*). Importantly, the levels of RDH10 mRNA did not display any significant changes (Fig. 1*B* and Fig. S1*B*). Taken together, these results strongly suggest that the decrease in the overall protein content of RDH10 observed in liver samples isolated from fasted mice (Fig. S7*B*) was due to some yet to be identified posttranscriptional mechanism(s). Furthermore, it is likely that the increase in RDH10 content associated with LD fractions reflects the enhanced biogenesis of LDs, which is characteristic of fasting as evidenced by the increase in perilipin 2 (ADRP) levels in LD fractions (Fig. 2*C*). However, the molecular mechanism responsible for targeting and association of RDH10 with LDs remains to be elucidated.

Previously, we reported that RDH10 operates in close coordination with DHRS3 (44, 45). To evaluate potential changes in expression and subcellular distribution of DHRS3 during the fed-to-fasted transition, we analyzed MT, MS, and LD fractions by western blot analysis using previously validated DHRS3 antibodies (44). As illustrated in Figure 2, *A*–*D* and Fig. S7*A*, in the fed state, the subcellular localization pattern of DHRS3 was similar to that displayed by RDH10, *i.e.*, DHRS3 protein was most abundant in MS fractions, followed by LD and MT fractions. In the samples prepared from fasted animals, DHRS3 content was significantly decreased in MS fraction, but it was increased in LDs (Fig. 2*E*). The combined content of DHRS3 in all membrane fractions appeared to be unchanged (Fig. S7*B*). Taken together, these data indicate that while RDH10 and DHRS3 proteins showed similar patterns of expression and subcellular distribution during the fed-to-fasted transition, the molecular mechanisms responsible for the changes in protein amount and subcellular localization of DHRS3 during the fed-to-fasted transition are somewhat different from those that regulate the behavior of RDH10.

### Retinol dehydrogenase and retinaldehyde reductase activities in subcellular fractions isolated from the livers of fed and fasted mice

**Figure 3:**
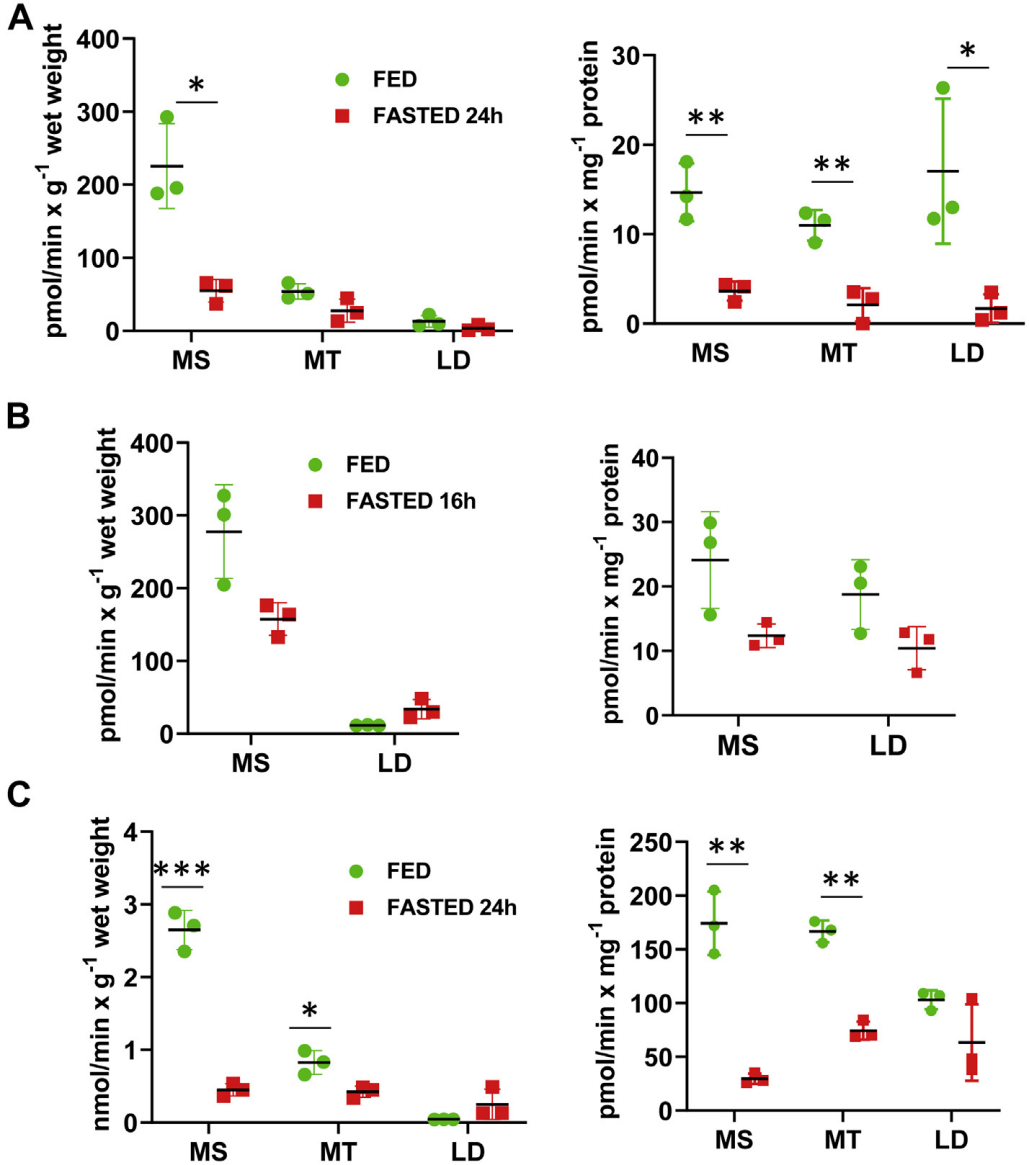
**Hepatic retinoid oxidoreductive activities in microsomal, mitochondrial, and lipid droplet membrane fractions from fed *versus* fasted mice.***A*, the NAD^+^-dependent retinol dehydrogenase activities of microsomes (MS), mitochondria (MT), and lipid droplets (LD) isolated from livers of 3-month-old fed or 24 h-fasted male mice (n = 3). The activity assays were performed with 100 μg of MS or MT protein for 15 min and with 30 μg of LD protein for 21 min. *B*, the NAD^+^-dependent retinol dehydrogenase activities of MS and LD isolated from the livers of 3-month-old fed or 16 h-fasted male mice (n = 3). The differences in activity rates did not reach statistical significance with *p* values of 0.06 to 0.08. *C*, the NADPH-dependent retinaldehyde reductase activity of MS, MT, and LD isolated from the livers of 3-month-old fed or 24 h-fasted male mice (n = 3). ∗*p* < 0.05; ∗∗*p* < 0.01; ∗∗∗*p* < 0.001. *Error bars*, S.D. NADPH, nicotinamide adenine dinucleotide phosphate hydrogen.

The results of western blot analysis (Fig. 2) suggested that fasting causes a substantial decrease in the overall hepatic content of RDH10 protein as well as changes in its subcellular distribution. To determine whether these changes affected the overall NAD^+^-dependent retinol dehydrogenase activity, we assayed the oxidation of retinol to retinaldehyde by MT, MS, and LDs isolated from the livers of fed and fasted mice. These measurements revealed that in the fed state, MS membranes accounted for the largest portion of the retinol dehydrogenase activity, followed by MT and LDs (Fig. 3*A*, *left*). The activity per milligram of membrane protein was comparable across all three membrane fractions (Fig. 3*A*, *right*). Importantly, all subcellular fractions isolated from the livers of mice fasted for 24 h displayed a decrease in retinol dehydrogenase activity. An approximately fourfold decrease was observed in the NAD^+^-dependent retinol dehydrogenase activity of MS and LD fractions and a twofold decrease in the activity of MT-associated membranes (Fig. 3*A*). The changes in retinol dehydrogenase activities of MS and LD fractions isolated from the livers of mice fasted for 16 h did not reach statistical significance (Fig. 3*B*).

Interestingly, based on the results of western blotting (Fig. 2), which show a much higher “density” of RDH10 per gram of LDs compared with MS and MT, it would be expected that LDs display a much greater retinol dehydrogenase activity per milligram of LD protein compared with MS and MT. Surprisingly, this is not the case. In fact, enhanced biogenesis of LDs in fasting appeared to be associated with further decrease in LD retinol dehydrogenase activity (Fig. 3*A*), in spite of the increase in RDH10 levels in LDs (Fig. 2). The reasons underlying the seemingly lower activity of RDH10 in LDs are unclear at this time. However, this might serve as one of the mechanisms responsible for the modulation of hepatic biosynthesis of RA. In this respect, it is interesting to note that recent studies suggest that during metabolic stresses, in addition to serving as nutrient reservoirs, LDs also function as storage sites for proteins (46). Therefore, it is feasible that LDs serve as a reservoir of a less active RDH10 that is created during metabolic stress associated with fasting.

RA homeostasis is also affected by the activity of DHRS3 and other retinaldehyde reductases, which convert retinaldehyde back to retinol in nicotinamide adenine dinucleotide phosphate hydrogen (NADPH)-dependent manner (47, 48). Our data showed that metabolic stress associated with fasting caused a decrease in DHRS3 protein (Fig. 2). Previously, we reported that DHRS3 operates as an NADPH-dependent retinaldehyde reductase when complexed with RDH10 (44, 45). In addition, in this study, we also observed a very significant decrease in the hepatic levels of mRNA for RDH11 (Fig. 1*B*). Recently, it was demonstrated that RDH11 acts as a hepatic NADPH-dependent retinaldehyde reductase (48). Taken together these observations indicate that fasting could cause a downregulation of hepatic retinaldehyde reductase activity along with downregulation of NAD^+^-dependent retinol dehydrogenase activity. To examine this possibility, we determined the NADPH-dependent retinaldehyde reductase activities of subcellular membranes isolated from the livers of fed and fasted mice. As shown in Figure 3*C*, this analysis revealed that, MS membranes contained the largest fraction of the NADPH-dependent retinaldehyde reductase activities followed by MT membranes. Importantly, these activities were significantly reduced in MS and MT membranes isolated from the livers of fasted animals. A sixfold decrease was observed in the NADPH-dependent retinaldehyde reductase activity of MS membranes and a twofold decrease in the activity of MT-associated membranes (Fig. 3*C*). In contrast, the contribution of LDs to the hepatic retinaldehyde reductase activity of fasted mice increased approximately sixfold compared with LDs isolated from fed mice (0.25 ± 0.2 *versus* 0.043 ± 0.002 nmol/min × g^−1^ wet weight) (Fig. 3*C*, left). This increase could be due to the overall expansion of LD protein fraction. The total hepatic retinaldehyde reductase activity was unchanged (Fig. S7*B*).

In the case of retinol dehydrogenase activity, the effects of fasting, at least in part, reflect the decrease in the cellular levels of RDH10, as well as DHRS3, because DHRS3 stimulates the retinol dehydrogenase activity of RDH10 when they form a complex (44). The decrease in retinaldehyde reductase activity in MS and MT can be explained by the decrease in protein levels of DHRS3 in MS and MT and also by the decrease in protein levels of RDH10, which is necessary for DHRS3 retinaldehyde reductase activity. Lastly, the decrease in hepatic retinaldehyde reductase activity might stem from the decrease in hepatic RDH11 levels that happen as a result of a decrease in its expression (Fig. 1*B*).

### Effects of fasting on hepatic retinoid levels

**Figure 4:**
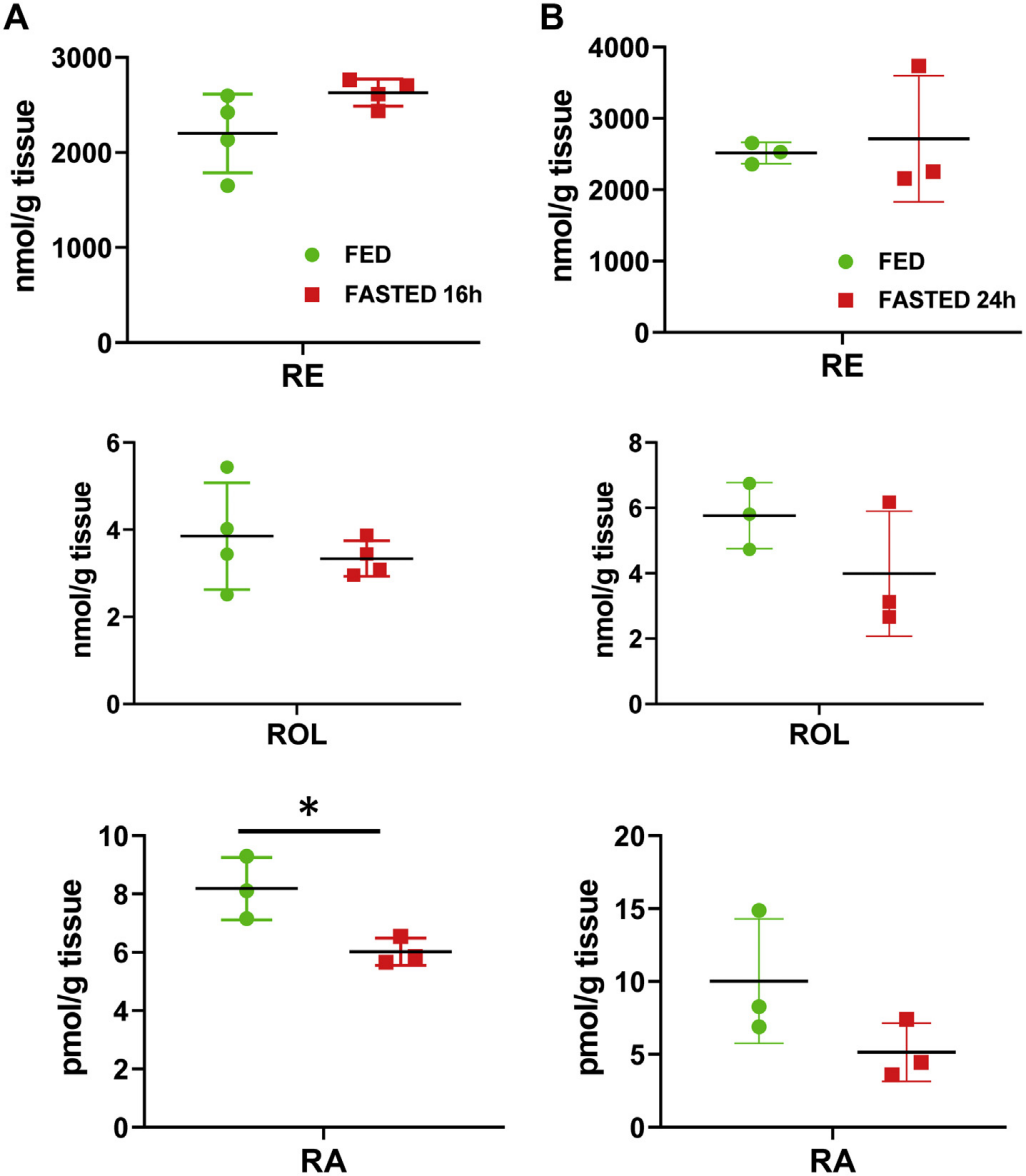
**Hepatic retinoid levels in fed *versus* 16 h-fasted (*A*) or 24 h-fasted (*B*) mice.** Livers from 3-month-old male mice were collected, homogenized, and extracted for analysis of retinyl esters (RE), retinol (ROL), and retinoic acid (RA). Retinoids were separated by reversed-phase HPLC and quantified using corresponding calibration curves. ∗*p* < 0.05. *Error bars*, S.D.

Analysis of expression and activities of retinol metabolizing enzymes described above suggested that fasting might affect the hepatic RA biosynthesis. To examine this possibility, we characterized the retinoid levels in the livers obtained from 3-month-old fed and 16 h-fasted male mice. Retinyl esters, retinol, and RA were extracted from the liver homogenates and their levels were determined using reversed-phase HPLC. As shown in Figure 4*A*, this analysis revealed that the levels of retinol and retinyl esters in fasted livers remained largely unchanged. Importantly, the hepatic RA levels demonstrated statistically significant decrease of approximately 1.4-fold (8.2 ± 1 pmol/g *versus* 6 ± 0.5 pmol/g, *p* = 0.03, n = 3). HPLC trace of the peak corresponding to RA and its spectral characteristics are shown in Fig. S8. In the livers of 24 h-fasted mice (Fig. 4*B*) the decrease in RA did not reach statistical significance. In conjunction with other results showing that fasting is associated with changes in RA-regulated transcriptome (Fig. 1*B*); the decreased abundance of RDH10 and DHRS3 proteins (Fig. 2); as well as the decrease in the NAD^+^-dependent retinol dehydrogenase activity in MT, MS, and LD fractions isolated from the livers of fasted mice (Fig. 3, *A* and *B*), the lower RA levels in 16 h-fasted livers suggest that metabolic stress and remodeling associated with fasting affect RA biosynthesis and signaling.

### Hepatic metabolic remodeling after 6 h of fasting

**Figure 5:**
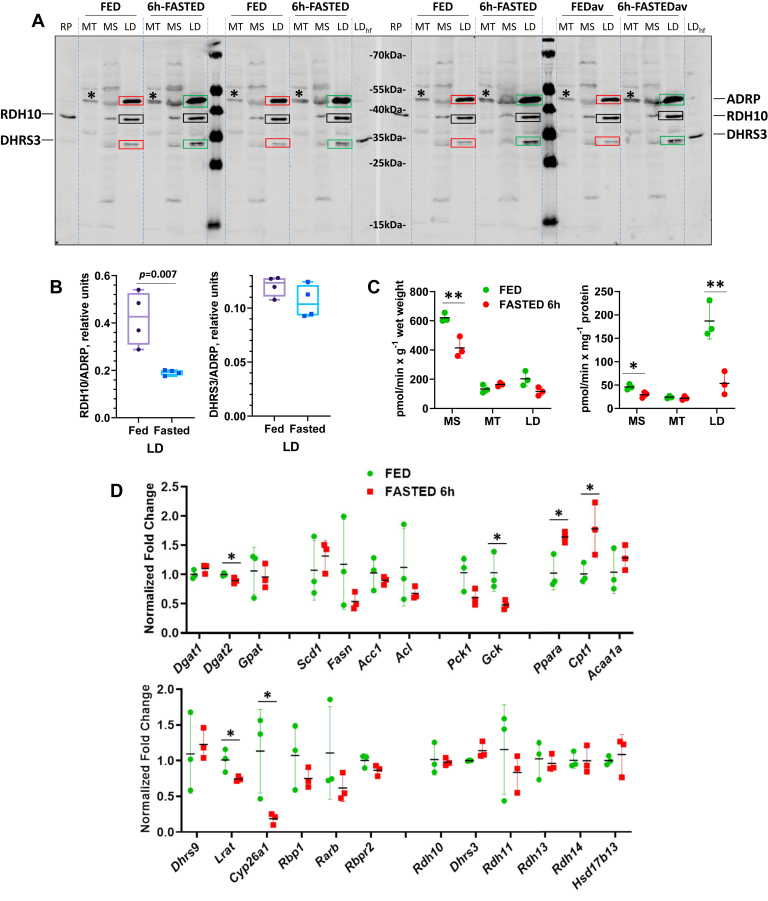
**Protein levels of RDH10 and DHRS3 in hepatic membrane fractions from fed *versus* 6 h-fasted mice.***A*, western blot analysis of RDH10 and DHRS3 protein content in mitochondria (MT), microsomes (MS), and lipid droplets (LD) isolated from 3-month-old male mice (n = 3). The loading was as described under Figure 2. The blots were incubated with a mixture of RDH10 and DHRS3 antibodies diluted 1:3000 each followed by incubation with Cy5 secondary anti-rabbit antibodies (1:2500). After developing the blots, they were incubated with ADRP (perilipin 2) antibodies diluted 1:3000. The *red boxes* in fed liver samples denote perilipin 2 (ADRP) and DHRS3 proteins that increase in LDs upon fasting (compare to *green boxes*). The unchanged RDH10 bands are enclosed in *black boxes*. *B*, relative quantities of RDH10 and DHRS3 proteins in lipid droplets from fed and 6 h-fasted mice. The blots were scanned using ImageQuant and the volume is presented as the total pixel intensity. *Error bars*, S.D. Abbreviations are: LDhf, lipid droplet fraction from the livers of mice on high-fat diet; RP, recombinant untagged human RDH10 and DHRS3 proteins overexpressed in HEK293 cells. The middle lane contains PageRulerTM Prestained Protein Ladder (Fermentas, catalogue #SM0671); ∗denotes a nonspecific protein band recognized by RDH10 antibodies in mitochondrial membranes. *C*, assays of NAD^+^-dependent retinol dehydrogenase activity using membrane fractions from fed and 6 h-fasted male mice. *D*, QPCR analysis of gene expression. ∗*p* < 0.05; ∗∗*p* < 0.01.

As discussed in the article by Ayala *et al.* (49), “In a typical metabolic study, mice are fasted for either 14 to 18 h (overnight fast) or for 5 to 6 h (morning fast).” The authors suggested that overnight fasting is useful for reducing variability in baseline blood glucose for studies where the focus is on glucose utilization. Otherwise, they considered a 5- to 6-h fast as being sufficient to assess insulin action within a more physiological context. To investigate whether the changes in subcellular localization of RDH10 and DHRS3 occur after a shorter fast, we collected the livers from C57BL/6J mice that were deprived of food for 6 h starting at 6 AM. The subcellular fractions were isolated immediately from fresh livers and purity of the fractions was verified by immunoblotting for markers of individual fractions (Fig. S6). Western blot analysis showed that perilipin 2 levels visibly increased in LD fractions of the livers from 6-h fasted mice (Fig. 5*A*), suggesting that LD fractions started to expand. Western blot analysis also showed an increase in DHRS3 levels in LDs. Interestingly, RDH10 levels displayed a small decrease in MS, but little or no changes in LD fractions. Quantification of RDH10 and DHRS3 protein levels relative to LD marker perilipin 2 showed a lower abundance of RDH10 in LDs but no change in abundance of DHRS3 after 6 h of fasting (Fig. 5*B*), suggesting that DHRS3 protein, but not RDH10, is increasing in LD simultaneously with perilipin 2. Thus, DHRS3 and RDH10 displayed notable differences in the timeline of their response during the adaptation to fasting.

In agreement with the data for 16 h and 24 h fasting, the NAD^+^-dependent retinol dehydrogenase activity of the total microsomal membrane fraction has decreased and the activity per milligram membrane protein has also decreased for MS and LD membranes (Fig. 5*C*). Importantly, qPCR analysis showed that metabolic remodeling of the liver was already under way after 6 h of fasting and this included significant changes in RA-regulated retinoid metabolic genes *Lrat* and *Cyp26a1* (Fig. 5*D*), expression of which was decreased, suggesting reduced RA signaling, in agreement with the apparently lower retinol dehydrogenase activity of microsomal membranes.

### Hepatic metabolic remodeling associated with T1D

A very extensive metabolic stress and remodeling are also associated with metabolic diseases such as T1D (50, 51). Taking into consideration our findings showing that fed-to-fasted transition causes significant changes in RA metabolism and signaling, we were interested in examining whether metabolic remodeling associated with T1D affects hepatic RA metabolism and signaling as well. Toward this goal, we analyzed an Ins2Akita mouse model in which a spontaneous mutation of *Ins2* gene results in early onset diabetes (52). The heterozygous Ins2Akita mice are a well-established model of T1D that develops hyperglycemia, insulinopenia, polydipsia, and polyuria as early as 3 to 4 months of age (53). Considering that only male Ins2Akita mice develop severe hyperglycemia, while females show much lesser pronounced responses due to the protective effects of estrogen (54, 55), all experiments described here were conducted on male mice at the age of 4 to 5 months.

**Figure 6:**
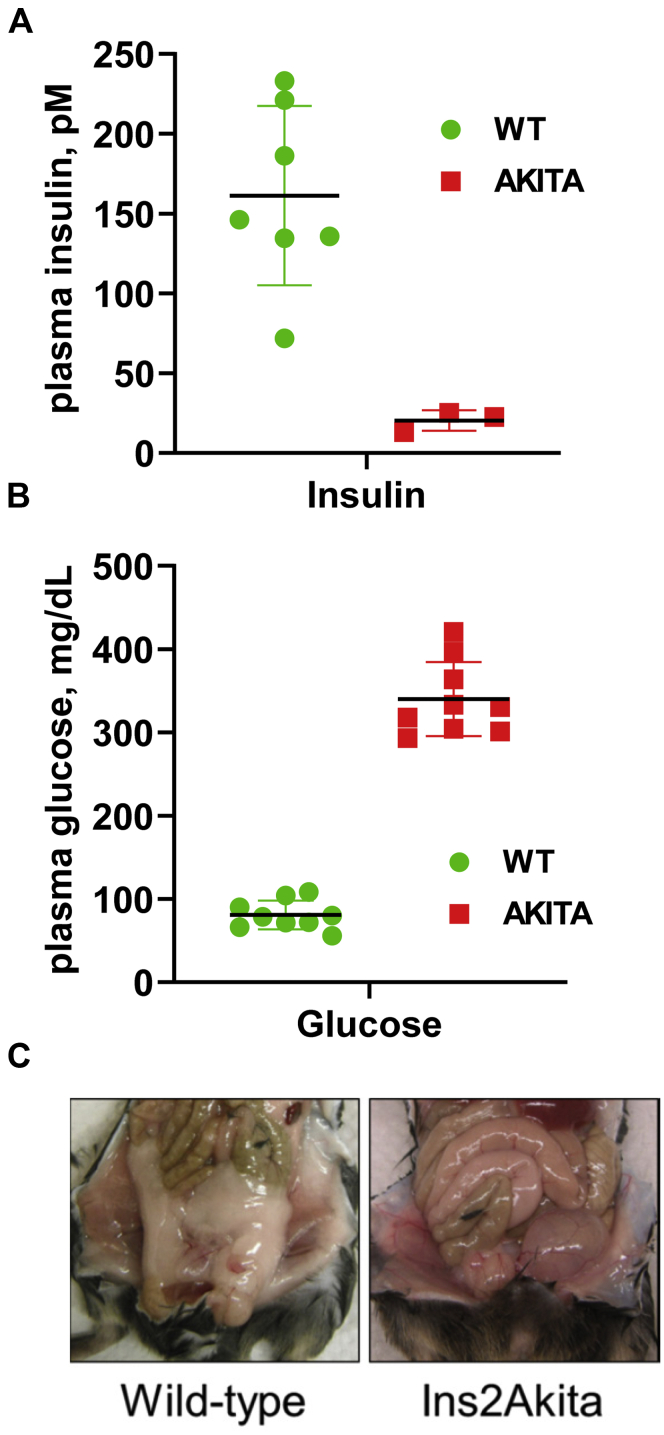
**Hepatic metabolism and phenotypic features of heterozygous Ins2Akita mice.***A*, plasma insulin in 3-month-old fed WT and heterozygous Ins2Akita mice. *B*, fasted blood glucose in 4- to 6-month old WT and Ins2Akita mice. *C*, fat depots in 4-month-old WT and Ins2Akita mice.

**Figure 7:**
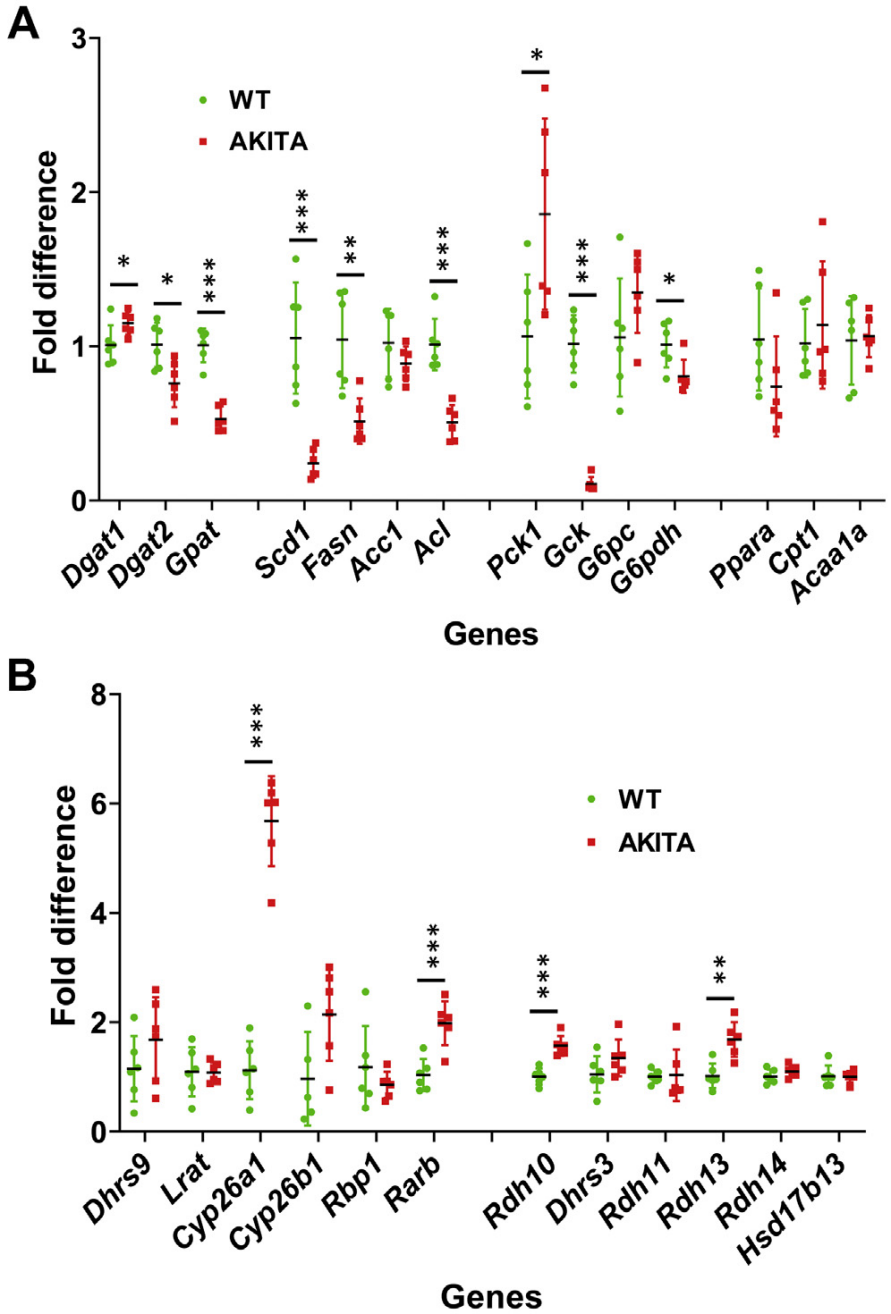
**Gene expression in the livers of Ins2Akita (AKITA) *versus* wild-type mice (WT).***A*, differences in expression of lipid and carbohydrate metabolic genes. *B*, differences in expression of retinoid metabolic genes and RA-regulated genes. QPCR analysis was performed using mRNA isolated from the livers of 6-month-old fed male mice (n = 6 for each group). ∗*p* < 0.05; ∗∗*p* < 0.01; ∗∗∗*p* < 0.001. *Error bars*, S.D.

As shown in Figure 6*A*, Ins2Akita mice displayed a significant decrease in circulating insulin levels already by the age of 3 months. By the age of 5 months, they developed a full-blown hyperglycemia (Fig. 6*B*) and showed a significant decrease in whole body fat mass (Fig. 6*C*), which happened as a result of enhanced lipolysis in adipose tissue. As illustrated in Figure 7*A*, analysis of the expression patterns of the major metabolic genes responsible for general hepatic metabolism in Ins2Akita mice using qPCR revealed that diabetic mice were gluconeogenic, as evidenced by the induction of *Pck1*, and had a severely reduced ability to buffer blood glucose (downregulation of *Gck*). The hepatic capacities for FA biosynthesis in Ins2Akita mice were significantly diminished as well (decrease in *Fasn*, *Acl*, *Scd1*). The capabilities to generate TAG were largely preserved, although *Dgat2* and, especially *Gpat*, decreased significantly (Fig. 7*A*). Together, these findings demonstrated an extensive metabolic remodeling associated with metabolic stress caused by T1D and were in agreement with earlier findings (50, 51, 52). To determine whether metabolic remodeling caused by T1D can affect the retinoid metabolism and signaling, we examined the expression levels of retinoid metabolic genes and genes regulated by RA by qPCR (Fig. 7*B*). This analysis revealed that liver samples from diabetic mice displayed a relatively small increase in transcript levels for *Rdh10*, *Rdh13*, and *Rarb* and an over fivefold increase in the level of mRNA for *Cyp26a1* (Fig. 7*B*). As discussed above, in the liver, *Cyp26a1* is believed to be primarily responsible for the disposal of hepatic RA (30, 31), and its expression is highly sensitive to RA levels (32, 33). Thus, the overexpression of *Cyp26a1* in the livers obtained from Ins2Akita mice suggests that metabolic stress and metabolic remodeling associated with T1D affect hepatic retinoid metabolism and signaling.

### Expression and subcellular localization of hepatic RDH10 and DHRS3 in WT and diabetic Ins2Akita mice

**Figure 8:**
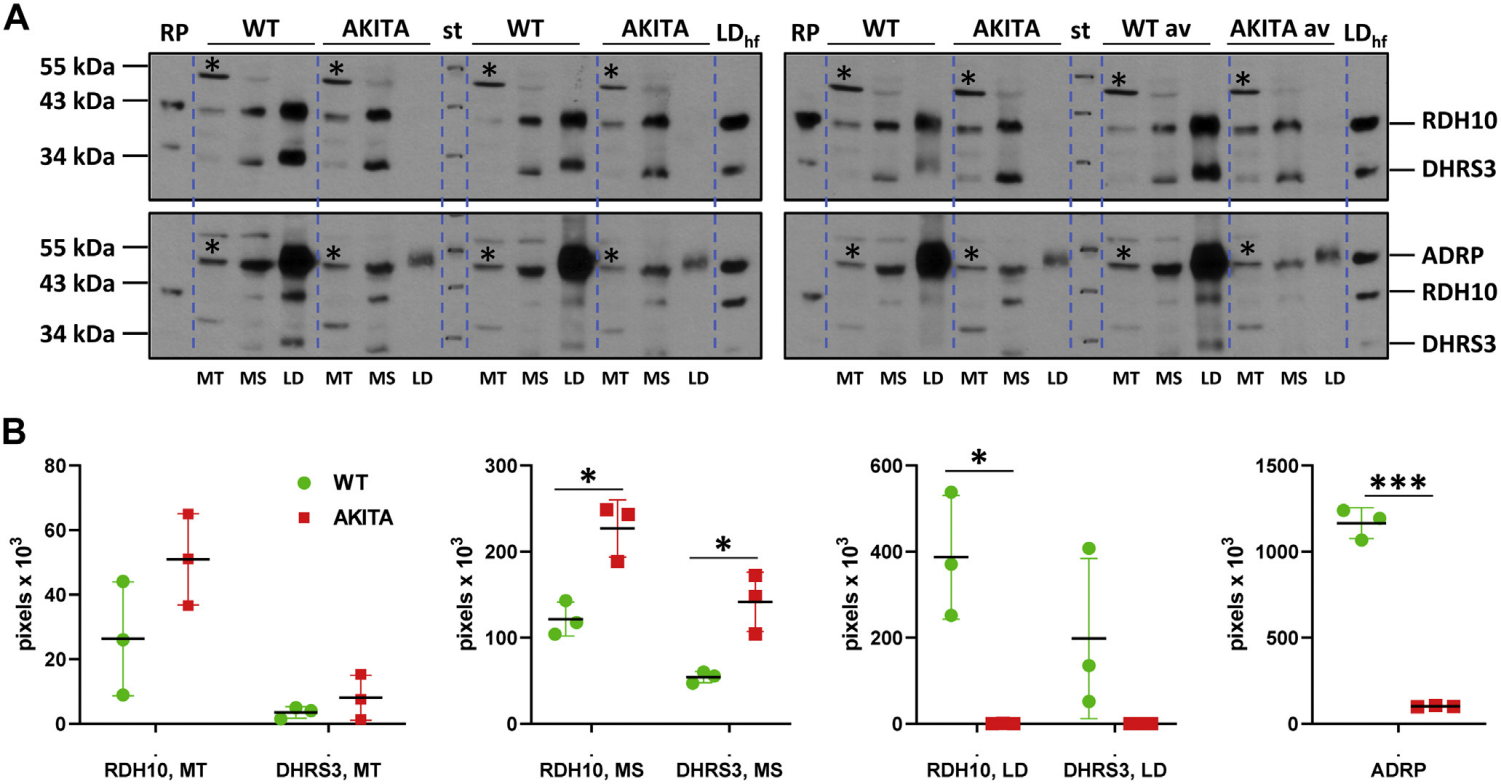
**Protein levels of RDH10 and DHRS3 in hepatic membrane fractions from wild-type *versus* Ins2Akita mice.***A*, western blot analysis of RDH10 and DHRS3 protein content in mitochondria (MT), microsomes (MS), and lipid droplets (LD). Each membrane fraction was resuspended in 200 μl, the MS and MT membrane fractions (but not LD fraction) were diluted fivefold and 10 μl of each fraction was loaded onto two separate gels. The last sample set represents the average of fractions from individual mice mixed together (WT av and AKITA av). The blot was first incubated with a mixture of RDH10 and DHRS3 antibodies, exposed to film (*upper panel*), and then incubated with ADRP antibodies (*lower panel*); *Asterisk* (∗) denotes a nonspecific band recognized by RDH10 antibodies in mitochondrial membranes. The results shown are from three individual wild-type C57BL/6J (WT) and three C57BL/6-Ins2Akita/J (heterozygous for Ins2Akita) male mice (Akita), all at 6 months of age. A representative Coomassie-stained gel and Ponceau-stained blot showing protein amount in each fraction is depicted in Fig. S5, *B* and *C*. *B*, quantification of RDH10 and DHRS3 proteins in hepatic membrane fractions from WT and Akita mice. The blots were scanned using UN-SCAN-IT and the data are presented as total pixel density. Abbreviations are: LD_hf_, lipid droplet fraction from the livers of mice on high-fat diet; RP, recombinant untagged human RDH10 and DHRS3 proteins overexpressed in HEK293 cells. ∗*p* < 0.05; ∗∗∗*p* < 0.001. *Error bars*, S.D.

Our studies of the fed and fasted mice demonstrated significant changes in cellular abundance and subcellular localization of RDH10, which was shown to be one of the major retinol dehydrogenases contributing to the biosynthesis of RA in mouse liver (41). Considering that analysis of RA target genes revealed overexpression of *Cyp26a1* (Fig. 7*B*), which is indicative of enhanced RA signaling, we investigated the possibility that the enhanced hepatic RA signaling in T1D might be due, at least in part, to the changes in expression and/or subcellular localization of RDH10 caused by metabolic stress and metabolic remodeling associated with T1D. Toward this goal, we examined the protein levels of RDH10 in MT, MS, and LD fractions isolated from the livers of control and diabetic mice using western blot analysis. As shown in Figure 8*A*, western blot revealed an increase in RDH10 protein content in MS and MT membrane fractions isolated from diabetic mice relative to wild-type (WT) mice. Quantification of the western blot data carried out as described under Experimental procedures showed that the content of RDH10 was 1.7-fold higher in MS fractions isolated from the livers of Ins2Akita mice compared with WT mice (Fig. 8*B* and Fig. S9*A*). On the other hand, LD fractions isolated from the livers of Ins2Akita mice displayed little if any RDH10 protein even after extended exposure times. This likely stems from the greatly decreased amount of LDs in the livers of Ins2Akita mice as evidenced by western blot analysis for perilipin 2 (ADRP) that showed a greatly decreased immunoreactivity for perilipin 2 in LD fractions from diabetic mice (Fig. 8*A*). The latter likely reflected dyslipidemia caused by T1D that we observed in Ins2Akita mice (Fig. 6*C*). Lastly, despite the clear differences in the distribution of RDH10 among the membrane fractions, the total amount of hepatic RDH10 protein was very similar between Ins2Akita mice and WT mice (Fig. S9*B*).

Considering that *in vivo* RDH10 operates in close cooperation with DHRS3, we employed western blotting to investigate the potential changes in the expression and subcellular localization of DHRS3 in samples isolated from control and diabetic mice (Fig. 8*A*). This analysis revealed that DHRS3 colocalized with RDH10 in MS and MT membrane fractions. Based on quantification of western blot data, DHRS3 abundance in MS fractions was increased (Fig. 8*B*). Similar to RDH10, DHRS3 protein was undetectable in LD fractions isolated from the livers of diabetic mice (Fig. 7*A*). Based on quantification data, the total amount of hepatic DHRS3 protein was very similar between Ins2Akita mice and WT mice (Fig. S9*B*). Thus, it appears that metabolic stress and remodeling caused by T1D in Ins2Akita mice trigger significant changes in subcellular distribution of RDH10 and DHRS3 proteins. This, in turn, might affect the cellular levels of NAD^+^-dependent retinol dehydrogenase activity and, thereby, contribute to the enhanced RA production.

### Retinol dehydrogenase and retinaldehyde reductase activities in hepatic subcellular fractions isolated from the livers of control and diabetic mice

**Figure 9:**
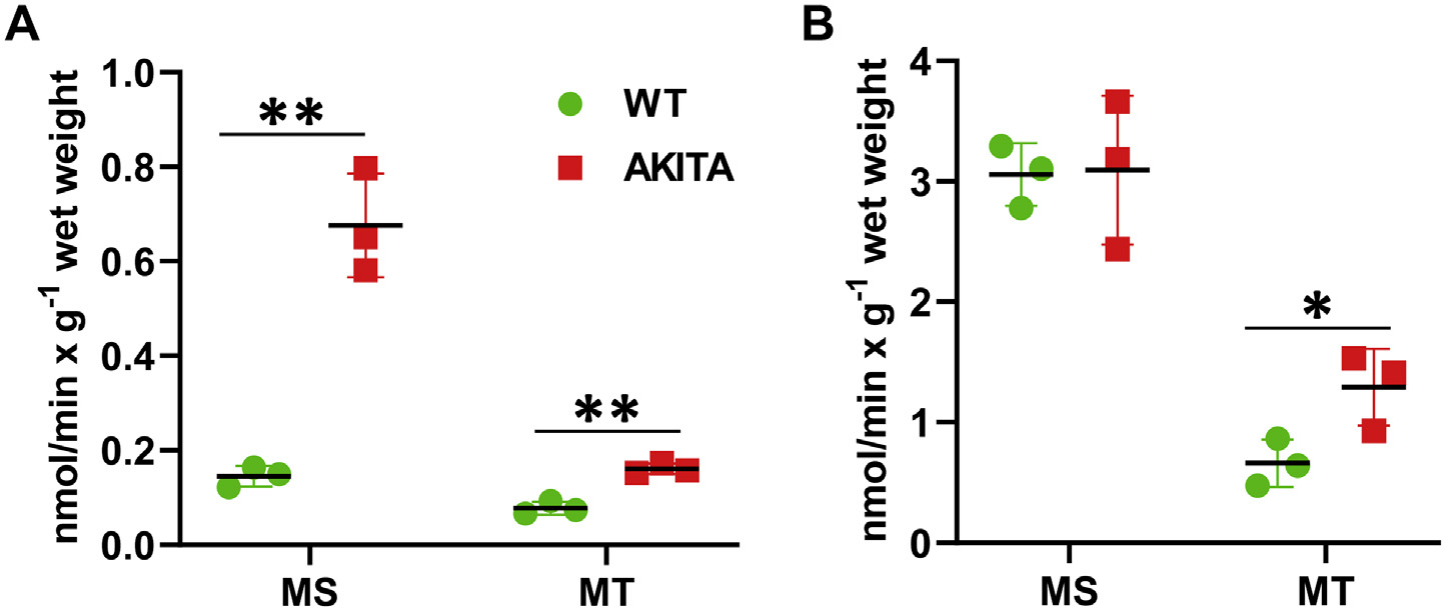
**Retinoid activities of subcellular membrane fractions in the livers of Ins2Akita *versus* wild-type mice.***A*, the NAD^+^-dependent retinol dehydrogenase activities of microsomal (MS) and mitochondrial (MT) fractions were normalized per g wet weight of liver samples. *B*, the retinaldehyde reductase activities of MS and MT fractions were normalized per g wet weight of liver samples. ∗*p* < 0.05; ∗∗*p* < 0.01. *Error bars*, S.D.

To further investigate the effects of metabolic stress and metabolic remodeling associated with T1D on biosynthesis of RA, we characterized the NAD^+^-dependent retinol dehydrogenase and NADPH-dependent retinaldehyde reductase activities in subcellular fractions isolated from the livers of WT control and Ins2Akita diabetic mice. As illustrated in Figure 9, these studies demonstrated that the NAD^+^-dependent retinol dehydrogenase activity of the total MS fraction and the total MT fraction isolated from the livers of diabetic mice was elevated by 4.5-fold and twofold, respectively (Fig. 9*A*). As would be expected from the results of western blot analysis, LD fractions obtained from diabetic Ins2Akita mice did not show an appreciable NAD^+^-dependent retinol dehydrogenase activity (data not shown). The NADPH-dependent retinaldehyde reductase activities of MS isolated from diabetic mice did not show any significant differences compared with those measured in MS fractions obtained from control mice, but MT activities were about twofold higher (*p* = 0.04) (Fig. 9*B*). Assays of LD fractions from diabetic mice did not display an appreciable NADPH-dependent retinaldehyde reductase activity similarly to the assays of NAD^+^-dependent retinol dehydrogenase activity (data not shown). Collectively, these observations lead us to conclude that T1D is associated with enhanced biosynthesis and, possibly, signaling of RA in the livers of affected animals.

## Discussion

Emerging evidence suggests that vitamin A status plays an important role in the regulation of enzymes involved in lipid and carbohydrate metabolism. For example, consumption of a vitamin A-deficient diet appears to result in hepatic glycogen deficiency associated with impaired gluconeogenesis (24), presumably due to low expression of PEPCK, a key gluconeogenic enzyme and a well-established target for regulation by RA (25, 26). Pharmacological RA dosing to mice fed a high-fat diet enhances lipid oxidation and inhibits lipid biosynthesis capacities in the liver (27). This raises an important question: what happens to hepatic RA levels and signaling during the fed-to-starved transition? It is generally believed that RA levels in adult tissues are maintained within a very narrow range specific for each type of cells (17, 18). Data presented here show that in the adult liver the rate of RA biosynthesis and the intensity of RA signaling vary over a wide range in accord with hepatic metabolic state.

During fasting, hepatic RA signaling appears to be decreased as indicated by the lower expression levels of RA-regulated genes. This finding is consistent with the 27% lower levels of hepatic RA in the livers of 16 h-fasted mice and with the decrease in the rate-limiting NAD^+^-dependent RDH activity associated with MS, MT, and LD membrane fractions. The MS membranes, which account for the largest fraction of the oxidative RDH activity, display a fourfold decrease in specific activity calculated per mg of microsomal protein (14.7 ± 3.2 *versus* 3.6 ± 1.1 nmol/min × mg^−1^). Interestingly, the decrease in microsomal activity correlates with the fourfold decrease in RDH10 protein in the microsomal fraction (230 ± 31 *versus* 57 ± 20 pixels × 10^3^). In addition to RDH10, the liver was shown to express another enzyme implicated in the oxidation of retinol to retinaldehyde, DHRS9 (SDR9C4) (41). However, DHRS9 appears to be a quantitatively minor contributor in the liver, with 22-fold lower expression level than RDH10 (41). Another enzyme than could potentially contribute to RDH activity in the liver is HSD17B13 (56). However, as reported recently, only human HSD17B13 displays a retinol dehydrogenase activity, whereas its murine ortholog is inactive toward retinol (57). Thus, so far, RDH10 appears as the best candidate for the role of a retinol dehydrogenase in the liver.

Previously, we reported that the flux of retinol to retinaldehyde through RDH10 is controlled through physical interaction of RDH10 with DHRS3 (44, 45). DHRS3 exhibits an NADPH-dependent retinaldehyde reductive activity when it is complexed with RDH10. Our data indicate that the overall NADPH-dependent specific activity of microsomes from fasted animals calculated per mg of microsomal protein is decreased 5.7-fold (0.17 ± 0.03 *versus* 0.030 ± 0.005 nmol/min × mg^−1^), which is generally consistent with the decrease in DHRS3 protein, but likely also includes the decrease in RDH11 (Fig. 1*B*), another NADPH-dependent retinaldehyde reductase. RDH11 is an integral membrane protein of endoplasmic reticulum, but it was not reported to be associated with LD. RDH11 is known to be regulated by sterol regulatory element-binding proteins, and its expression and hepatic protein levels are decreased during fasting (58).

A very different picture is presented by the lipid droplets. The protein amount of RDH10 in lipid droplets is increased upon starvation (375 ± 48 *versus* 512 ± 29 pixels × 10^3^). However, as discussed under *Results*, the oxidative RDH activity of lipid droplets is decreased fourfold. These data suggest that the specific activity of RDH10 in lipid droplets is lower than in microsomes or mitochondria. Similarly to RDH10, the abundance of DHRS3 in lipid droplets is increased upon fasting from 157 ± 38 to 359 ± 15 pixels x 10^3^. However, in contrast to the NAD^+^-dependent retinol dehydrogenase activity, the NADPH-dependent retinaldehyde reductase activity per g LD protein remains unchanged. Whether these differences in activities are associated with altered conformation/oligomerization of each enzyme in the monolayer leaflet of LD membrane as opposed to bilayer leaflets of endoplasmic reticulum membranes is currently unknown.

While RA signaling is decreased in the livers of fasted mice, it appears to be increased in the livers of mice with T1D as indicated by the upregulated expression of *Cyp26A1*. Western blot analysis shows a substantial increase in the RDH10 protein content in microsomal membranes from Ins2Akita mice, which correlates with the increase in total RDH activity. The specific NAD^+^-dependent retinol dehydrogenase activities of microsomal and mitochondrial membranes calculated per mg protein are fourfold greater for microsomal membranes isolated from the livers of Ins2Akita mice than in WT mice (42 ± 7 *versus* 12 ± 1 pmol/min × mg^−1^) and twofold greater for mitochondrial membranes (22 ± 2 *versus* 10 ± 1 pmol/min × mg^−1^). Thus, the increase in RA signaling is consistent with elevated RDH activities.

Collectively, our data indicate that the total amount of RDH10 protein in the membrane fractions isolated from the livers of fed, fasted, or diabetic mice strongly correlates with the overall RDH activity in these membrane fractions, suggesting that: (1) RDH10 acts as a major RDH in the liver; and (2) RDH10 activity is regulated through the changes in its protein abundance.

The lack of or insufficient insulin signaling in T1D keeps the liver in a chronic gluconeogenic and ketogenic mode even when dietary glucose is delivered from the gut. Interestingly, elevation of hepatic VA contents in patients with diabetes was observed in 1937 (59). The results of this study suggest that T1D has a profound effect upon the composition of hepatic subcellular fractions. Judging by perilipin 2 staining, which is the major structural component and biological marker of LD (60), T1D drastically depletes the LD fraction. Based on our data, LD plays an important role in the regulation of abundance and activity of RDH10 and DHRS3, which appear to act as major regulators of RA status in the adult liver, governing metabolic remodeling through changes in expression levels of key metabolic enzymes. Importantly, our data obtained with the livers of animals fasted for 6 h suggest that the changes in RDH10 levels and the RDH activity of membrane fractions occur during the early stages of adaptation to fasting, supporting the relevance of retinoid metabolism and RA signaling for the regulation of metabolic transition from the fed to fasted state.

## Experimental procedures

### Mice

Hepatic metabolic remodeling during the fed-to-fasted transition was studied using WT C57BL/6J mice obtained from Jackson Laboratory. Mice were housed in an AALAC-approved pathogen-free facility. For studies targeting metabolic interrelationships in fed state, mice were housed at 23 °C ± 2 deg. C with free access to water and food (standard rodent chow diet obtained from Harlan, catalogue number 7017) with a light cycle of 12 h light and 12 h dark. For 24 h-fasted group, food was withheld at the end of dark cycle. For 16 h-fasted group, food was withheld at 4 PM. For 6 h-fasted group, food was withheld at 6 AM. During fast, mice received unrestricted access to water. All studies were conducted with approval of Institutional Animal Care and Use Committee of the University of Alabama at Birmingham School of Medicine.

For biochemical analyses, the livers from fed and fasted mice were rapidly removed and cut into pieces. Liver samples for isolation of RNA and HPLC analysis were frozen at the temperature of liquid nitrogen and stored at −80 °C. Liver samples for isolation of membrane fractions were homogenized and subjected to differential centrifugation immediately after dissection or were frozen in liquid nitrogen and stored at −80 °C. We did not notice any differences in the purity of fractions isolated from fresh or frozen liver samples. Nevertheless, fresh liver samples were used for isolation of fractions from 16 h- and 6 h-fasted mice.

To validate the specificity of RDH10 antibodies, RDH10 null mutant as well as heterozygous and WT littermate embryos were generated and collected at E10.5 as previously described (38). Embryos were snap frozen in liquid nitrogen and kept at −80 °C until analyses.

To study the effects of T1D, C57BL/6J WT and C57BL/6-*Ins2*^*Akita*^/J mice heterozygous for spontaneous mutation *Ins2*^*Akita*^ were purchased from Jackson Laboratory. Groups of WT and Akita mice were housed under standard conditions of 12 h light/12 h dark cycles with temperature maintained at 23 °C ± 2 deg. C and fed *ad libitum* standard rodent chow diet (Harlan; no.7017). Insulin levels in Akita mice and their WT littermates were monitored using Ultra Sensitive Mouse Insulin ELISA Kit (Crystal Chem Inc, catalogue number 90080), following manufacturer’s instructions. Their blood glucose levels were monitored using Glucose Assay Kit (Abcam) as described above. Livers from WT and diabetic mice were rapidly removed, freeze-clamped with Wollenberger tongs at the temperature of liquid nitrogen, and stored at −80 °C for further analysis.

### Isolation of hepatic membrane fractions

Microsomes, mitochondria, and lipid droplets were isolated by differential centrifugation in sucrose gradient. Briefly, 200 mg of frozen liver tissue samples (or fresh livers from 16 h-fasted and 6 h-fasted mice taken immediately after sacrificing) was homogenized in 1 ml of ice-cold isolation buffer (0.25 M sucrose in PBS supplemented with 1 mM EDTA and protease inhibitors: 1 μg/ml aprotinin, 1 μg/ml leupeptin and 1 μg/ml pepstatin A) by Politron in three bursts for 10 s each on ice. Crude homogenates were further homogenized using 20 strokes in a glass homogenizer on ice. Samples were centrifuged at 3,000*g* for 10 min at 4 °C. The supernatants were removed into prelabeled 2 ml tubes for isolation of mitochondria; the pellets were washed with 300 μl of buffer and recentrifuged at 3,000*g* for 5 min. The supernatants from both centrifugations were combined in 2 ml tube.

The 3,000*g* supernatants were centrifuged at 10,000*g* for 10 min at 4 °C. The mitochondrial pellets were resuspended in 200 μl of reaction buffer (90 mM K_2_HPO_4_/KH_2_PO_4_, 40 mM KCL), supplemented with 20% glycerol and 1 mM EDTA. The supernatants were carefully layered onto 1 ml cushions of 0.6 M sucrose in PBS in chilled 5 ml centrifuge tubes. On top of hepatic 3,000*g* supernatants was layered reaction buffer (90 mM K_2_HPO_4_/KH_2_PO_4_, 40 mM KCL) to fill up the tube. The tubes were centrifuged at 105,000*g* for 90 min at 4 °C.

Lipid droplets were carefully collected by 200 μl wide-offset tips (wetted in reaction buffer to prevent LDs from sticking to the tip) in a minimal volume of reaction buffer and transferred to 1.5 ml Eppendorf tubes. If the volume of collected LD-containing fraction exceeded 0.5 ml, the sample was recentrifuged at 10,000*g* for 5 min at 4 °C and the buffer underlying the LD fraction was gently aspirated with a gel-loading tip, trying not to disturb the top LD layer left in 200 μl.

The microsomal pellets were washed with 200 μl of Isolation buffer, making sure not to disturb them. The wash buffer was discarded and pellets were resuspended using wide-tipped tips in 200 μl of Reaction buffer (90 mM K_2_HPO_4_/KH_2_PO_4_, 40 mM KCL), supplemented with 20% glycerol and 1 mM EDTA, and transferred to prelabeled 1.5 ml Eppendorf tubes. After the addition of dithiothreitol to all fractions to the final concentration of 1 mM, the tubes were flash frozen in liquid nitrogen and stored at −80 °C.

### Western blot analysis

Protein concentrations were determined according to Peterson’s modification with BSA as a standard (61). Equal parts of microsomal, mitochondrial, and lipid droplet fractions were separated by SDS-PAGE using standard Laemmli system with 12% separating gel and 4% stacking gel (mini gel, 140 V). The gels were transferred to nitrocellulose membrane for 1 h using semi-dry transfer unit, followed by blocking in 5% BSA in TBST for 1 h at room temperature.

Blots were probed overnight in cold room with the following primary antibodies: rabbit RDH10 antibodies at a 1:3000 dilution (Proteintech, catalogue number 14644-1-AP); rabbit DHRS3 antibodies at a 1:3000 dilution (Proteintech, catalogue number 15393-1-AP); rabbit ADRP/Perilipin 2 antibodies diluted 1:3000 (Proteintech, catalogue number 15294-1-AP); rabbit Cytochrome P450 Reductase antibodies diluted 1:4000 (Chemicon International, catalogue number AB1257); rabbit PDC (Pyruvate Dehydrogenase Complex) antibodies diluted 1:5000. Secondary goat anti-rabbit antibodies conjugated with horseradish peroxidase were obtained from Bio-Rad and used at a working dilution of 1:10,000. ECL Plex goat anti-rabbit Cy5 antibodies from Sigma were used at a 1:2500 dilution. Immunoreactive bands were visualized using the Pierce ECL Western Blotting Substrate (Thermo Scientific, catalogue number 32106), following the manufacturer’s recommendations. Blots were quantified with UN-SCAN-IT software (Silk Scientific Inc).

### qPCR analysis

For analysis of gene expression in the livers, ∼15 mg of frozen liver tissue was homogenized with TRIzol reagent (Ambion, Cat No. 15596-018) and RNA was extracted following the manufacturer’s protocol. The concentration of extracted RNA was determined using Nanodrop ND-1000 spectrophotometer (Thermo Scientific). First-strand cDNA was synthesized from 2.0 μg of total RNA with Superscript III first-strand synthesis kit (Invitrogen) according to the manufacturer's protocol. For real-time RT-PCR reactions, the cDNA was diluted 15-fold. Sequences of the primers are available by request. Real-time PCR analysis was conducted on Roche LightCycler480 detection system (Roche Diagnostics) with SYBR Green as probe (LightCycler480 CYBR Green I Master, Roche). Relative gene expression levels were calculated using the comparative Ct method by normalization to geometric mean of expression levels of four housekeeping genes (GAPDH, β-actin, m18S, and Hprt1). Unpaired *t*-test was used to test for statistical significance.

### Analyses of hepatic retinoid content

Frozen liver samples were homogenized in 1 ml of PBS on ice in the dark. For the analysis of RA, 1 ml aliquot of the homogenate was mixed with 1 ml ethanol containing 0.025 N KOH and extracted four times with 5 ml of hexane. Organic phase containing neutral retinoids was discarded; aqueous phase was acidified to pH below 2.0 by the addition of 0.045 ml of 4N HCl and extracted with 5 ml of hexane (62). Hexane layer was collected, dried under flow of nitrogen, and the residue was dissolved in 0.12 ml of 80:20 mix of solvent A (acetonitrile:2% (w/v) ammonium acetate:glacial acetic acid:methanol, 79:16:3:2) and acetonitrile. For the analysis of retinol and retinyl esters, 0.05 ml-aliquots of liver homogenate were mixed with 0.45 ml of PBS, mixed with 0.5 ml of ethanol, and extracted twice with 2 ml of hexane. Hexane layers from two extractions were combined and dried. The dry residue was dissolved in 0.1 ml of 70:30 mix of acetonitrile and dichloromethane. Internal standards in ethanol (10 μl of 1 μM acitretin for retinoic acid and 10 μl of 10 μM retinyl acetate for retinyl esters and retinol) were added to the homogenates before extractions.

For analysis of RA, 0.1 ml samples were separated using reverse-phase HPLC with SUPELCOSILSuplexpKb-100 column (Sigma-Aldrich) as a stationary phase and an isocratic mobile phase consisting of solvent A at 0.7 ml/min. Retinol and retinyl esters (0.03 ml) were separated by a gradient mobile phase at 0.7 ml/min as follows: 0 to 20 min, 100% solvent A; 20 to 21 min, change to 100% solvent B (acetonitrile:dichloromethane, 90:10); 21 to 45 min, 100% solvent B; 45 to 46 min, change to 100% solvent A; 46 to 50 min, 100% solvent A.

Separation was performed using Waters Alliance 2695 Separation Module and 2996 Photodiode Array Detector. Retinoids were identified by reference absorbance spectra and coelution with standards. Absorbance peak areas for each retinoid (extracted at 325 nm for retinol and retinyl esters, 357 nm for RA) were converted to pmol amount using linear regression of peak areas obtained by injections of serial dilutions of retinoid standards.

### Analysis of retinol dehydrogenase activity in hepatic membrane fractions

Samples of subcellular fractions (100 μg of protein for mitochondrial and microsomal fractions, and 30 or 40 μg for LD) were incubated with 3 μM all-*trans*-retinol or all-*trans*-retinaldehyde (Toronto Research Chemicals, Toronto, Canada) solubilized with bovine serum albumin as described (63) and 1 mM NAD^+^ or NADPH (Sigma-Aldrich) in 0.5 ml of the reaction buffer for 15 min (MT and MS) or 20 min (LD) at 37 °C. Reactions were stopped by the addition of equal volume of ice-cold methanol, and retinoids were extracted twice with 2 ml of hexane. Hexane layers were dried, and the dry residue was reconstituted in 0.2 ml of hexane:ethyl acetate (90:10). Retinoids were separated by normal-phase HPLC using Spherisorb S3W column (4.6 mm × 100 mm; Waters) and isocratic mobile phase consisting of hexane:ethyl acetate (90:10) at 1 ml/min and analyzed as described (63).

### Statistical analysis

Statistical significance was determined using two-tailed unpaired *t*-test.

## Data availability

All relevant data are contained within this article and in the supporting information.

## Conflict of interest

The authors declare that they have no conflicts of interest with the contents of this article.
