# Supplementary material for: Changes in retinoid metabolism and signaling associated with metabolic remodeling during fasting and in type I diabetes
Source: J Biol Chem. 2021 Jan 22;296:100323. doi: 10.1016/j.jbc.2021.100323 (PMC7949101; doi:10.1016/j.jbc.2021.100323)
Supplement: Supplemental Figures S1–S9 [file mmc1.pdf]

SUPPORTING INFORMATION

Changes in retinoid metabolism and signaling associated with metabolic remodeling during fasting and in type I diabetes.

Alla V. Klyuyeva,[#] Olga V. Belyaeva,[#] Kelli R. Goggans,[#] Wojciech Krezel[$], Kirill M. Popov,[#]* Natalia Y. Kedishvili[#]*

Table of Contents

Figure S1. Expression of housekeeping genes in fed and 24h-fasted livers.

Figure S2. Hepatic gene expression levels in fed *versus* 24 h-fasted mice.

Figure S3. Validation of RDH10 antibodies.

Figure S4. Validation of subcellular fractions.

Figure S5. Representative Ponceau-stained blots (A and C) and SDS-PAGE gel (B) showing total amount of protein in lanes containing mitochondrial (MT), microsomal (MS), and lipid droplet membranes (LD).

Figure S6. Comparison of protein levels of subcellular markers between hepatic fractions from fed and fasted mice.

Figure S7. Relative protein levels of RDH10 and DHRS3 in hepatic subcellular fractions of fed *versus* 24 h-fasted mice.

Figure S8. Detection of all-*trans*-retinoic acid in liver samples.

Figure S9. Relative protein levels of RDH10 and DHRS3 in hepatic subcellular fractions of WT *versus* Akita mice.

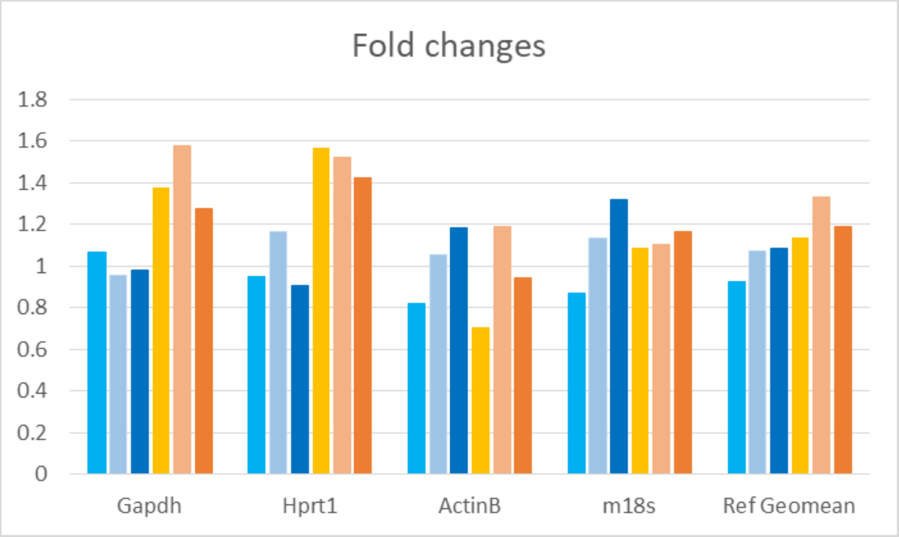

Figure S1. Expression of housekeeping genes in fed and 24h-fasted livers. Fed samples from 3 mice are shown in shades of blue; fasted samples from 3 mice are shown in shades of orange. While individual genes showed changes in expression levels, the geometric mean (Ref Geomean) of all four genes' expression was more stable and was used for normalization.

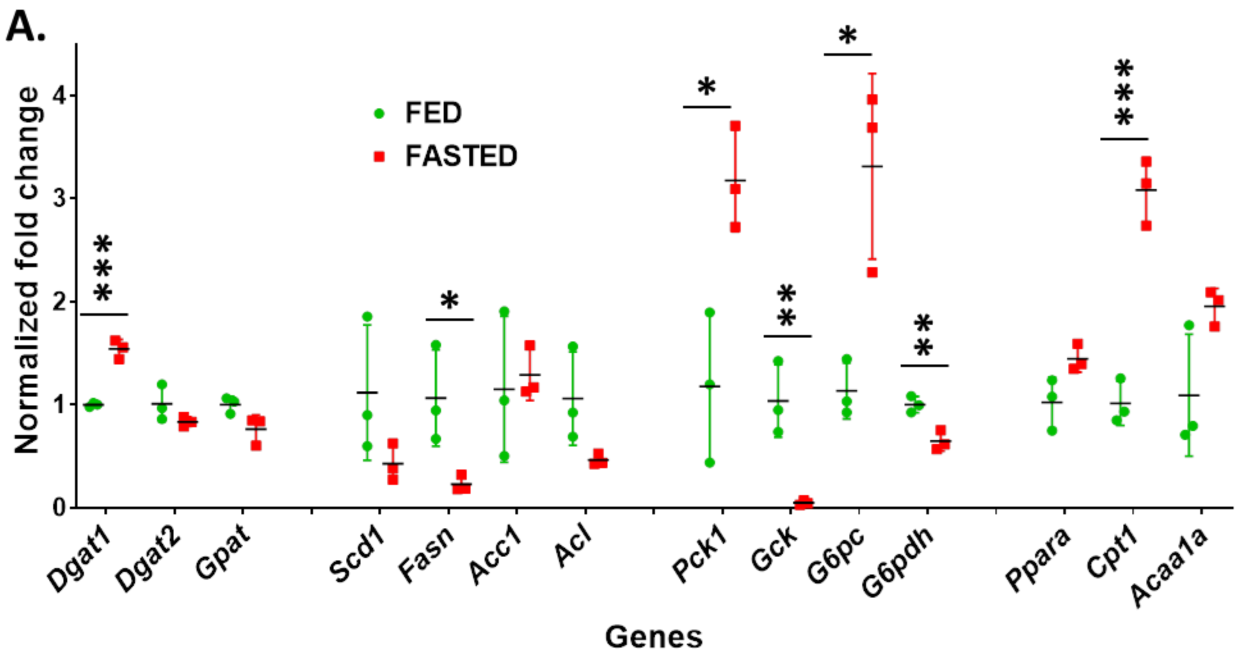

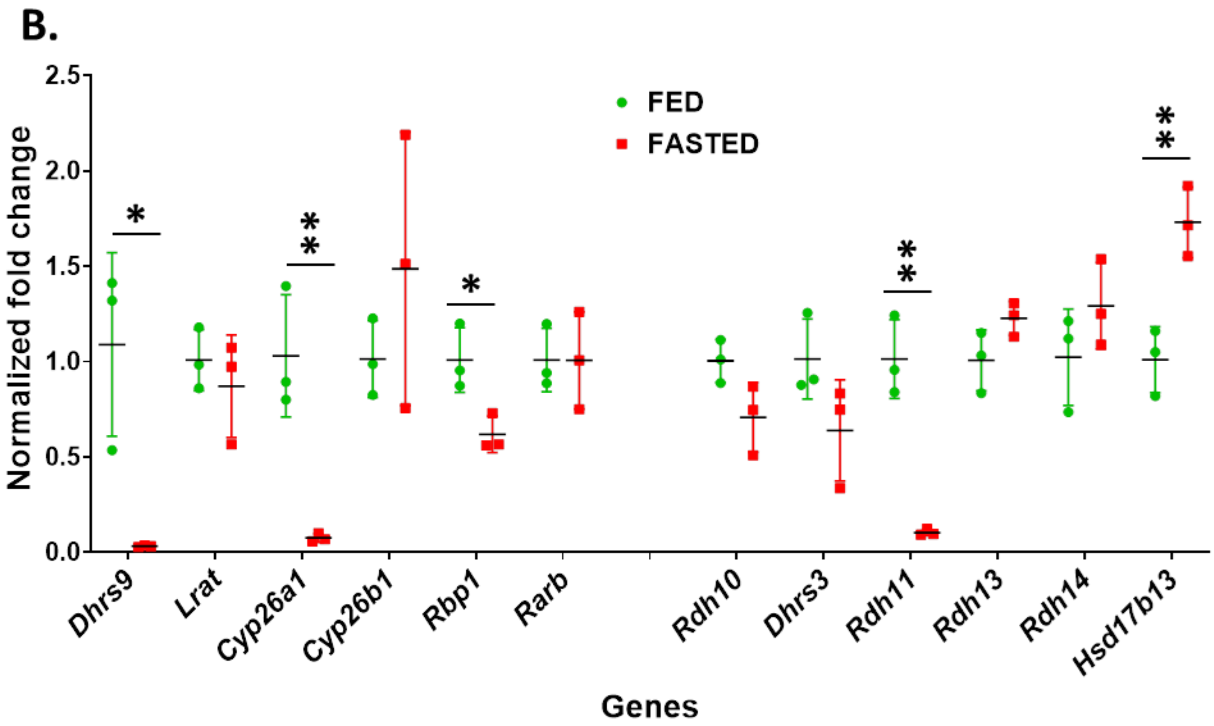

Figure S2. Hepatic gene expression levels in fed *versus* 24 h-fasted mice. A. Expression of lipid and carbohydrate metabolic genes. **B.** Expression of retinoid metabolic genes and RA regulated genes. qPCR analysis was performed using mRNA isolated from livers of 3-month old fed or 24 h-fasted male mice (n=3 for each group). *, $p < 0.05$; **, $p < 0.01$; ***, $p < 0.001$. *Error bars*, S.D.

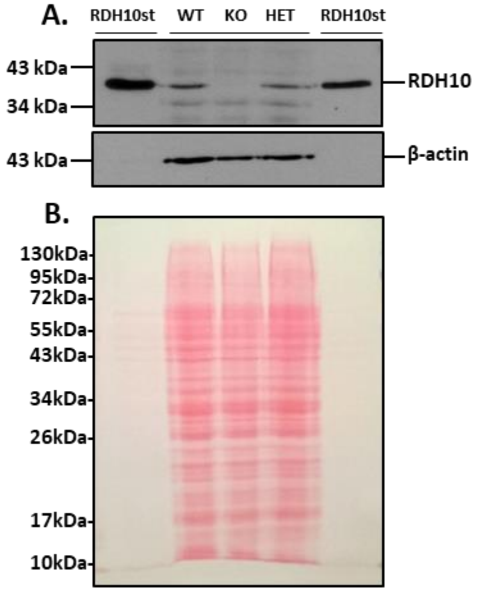

Figure S3. Validation of RDH10 antibodies. A. Western blot analysis of RDH10. Loading is as follows: lane 1, whole cell lysate of HEK293 cells overexpressing RDH10 (3 µg); lane 2, microsomal membranes isolated from wild-type mouse embryos (50 µg); lane 3, microsomal membranes isolated from RDH10 gene knockout mouse embryos (50 µg); lane 4, microsomal membranes isolated from RDH10 heterozygous mouse embryos (50 µg); lane 5, whole cell lysate of HEK293 cells overexpressing RDH10 (1.5 µg). Note the absence of immunostaining in microsomes isolated from RDH10 gene knockout embryos and the weaker immunostaining in microsomes isolated from RDH10 heterozygous embryos (HET), in agreement with the lower abundance of RDH10. **B. Protein loading.** The blot was stained with Ponceau to assess the protein loading and transfer before incubation with antibodies.

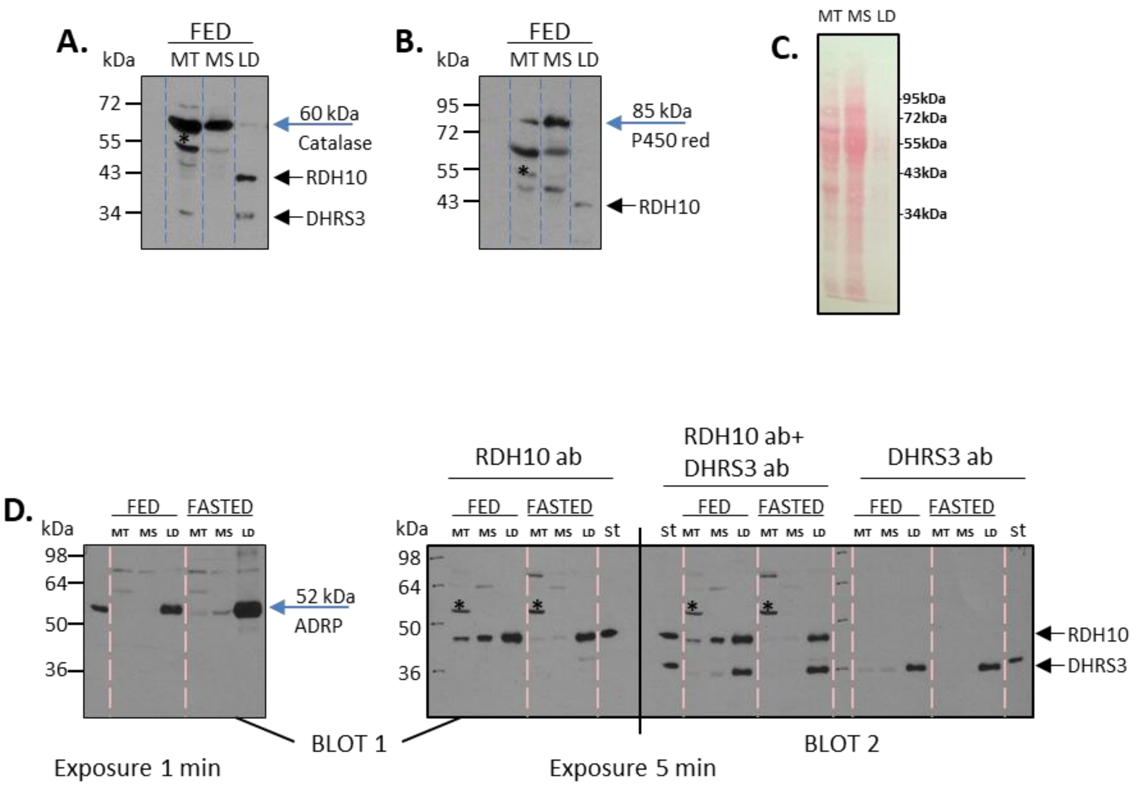

Figure S4. Validation of subcellular fractions. Heavier membrane fractions associated with mitochondria (MT), lighter membrane fractions associated with microsomes (MS) and floating membrane fractions associated with lipid droplets (LD) were isolated from frozen liver samples obtained from wild-type mice fed chow diet or fasted for 24 hours. **A.** The membrane was first incubated with a mixture of RDH10/DHRS3 antibodies as described under *Experimental Procedures* and then re-probed with antibodies against catalase (1:2,000, Abcam, catalog number ab16731). Catalases are mostly located in peroxisomes and mitochondria as both soluble and membrane-bound forms. **B.** The same membrane was then probed with antibodies against cytochrome P450 reductase (1:4,000, Chemicon International, catalogue number AB1257), which serves as a marker of microsomal membranes. **C.** Ponceau staining of the membrane corresponding to blots in A and B. Mitochondrial and microsomal fractions were diluted 1:5 whereas LD fractions were used as is undiluted and 12.5 µl of each fraction was loaded onto the gels. **D.** Characterization of antibodies against perilipin 2, RDH10 and DHRS3. Hepatic MT, MS and LD membranes isolated from fed or fasted mice and microsomes (1 µg) containing recombinant RDH10 and DHRS3 co-expressed in HEK 293 cells (st) were separated in two gels and transferred to nitrocellulose membranes. One half of each blot was incubated either with antibodies against perilipin 2 (ADRP) (1:3,000, Proteintech, catalogue number 15294-1-AP), the marker for lipid droplets; with antibodies against RDH10; with antibodies against RDH10 mixed with antibodies against DHRS3; or DHRS3 antibodies only. Asterisks (*) denote a non-specific band running at the level of ADRP, which originates from the incubation with RDH10 antibodies.

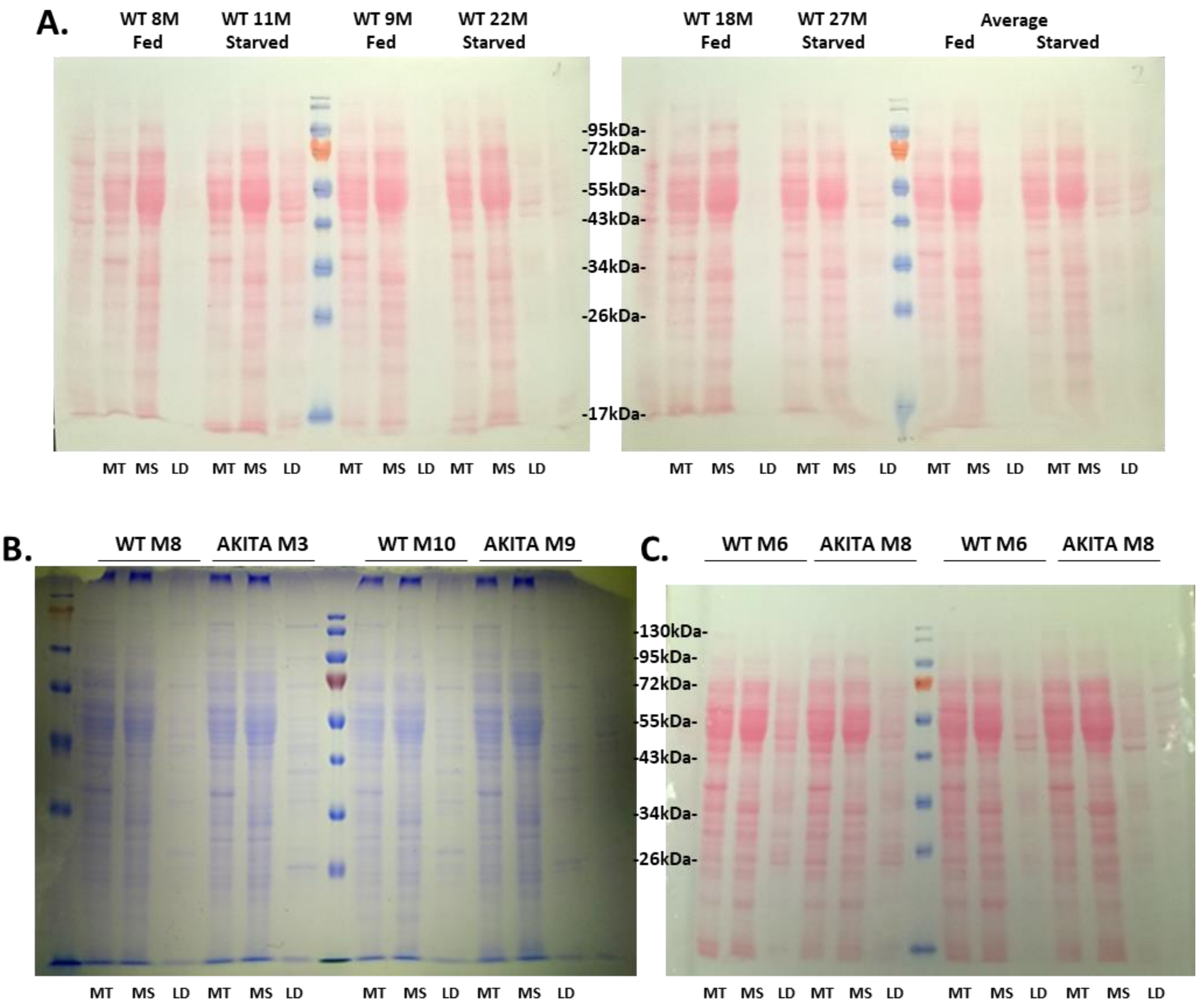

Figure S5. Representative Ponceau-stained blots (A and C) and SDS-PAGE gel (B) showing total amount of protein in lanes containing mitochondrial (MT), microsomal (MS), and lipid droplet membranes (LD). A. Blots corresponding to western blots for fed and fasted mice in Fig. 2 D. Each membrane fraction was resuspended in 200 µl, the microsomal and mitochondrial membrane fractions (but not lipid droplet fraction) were diluted 5-fold and 10 µl of each fraction were loaded onto 2 separate gels. The last sample set represents the average of fractions from individual mice mixed together. **B** and **C**. Membrane fractions from three individual wild-type C57BL/6J (WT) and three C57BL/6-Ins2Akita/J (heterozygous for Ins2Akita) male mice (AKITA).

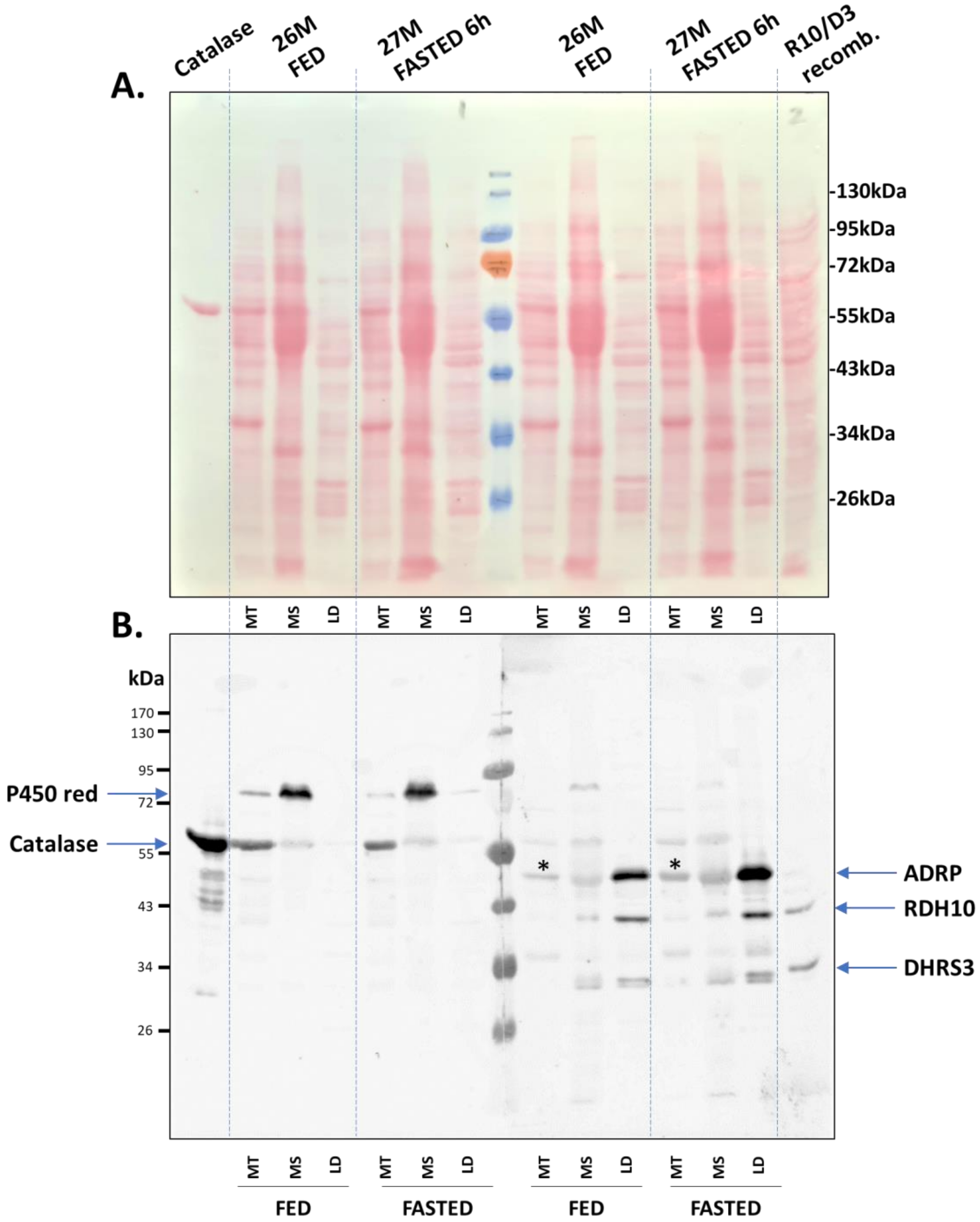

Figure S6. Comparison of protein levels of subcellular markers between hepatic fractions from fed and fasted mice. A. Ponceau-stained blot showing protein loading. R10/D3 denotes RDH10 and DHRS3 proteins co-expressed in HEK293 cells. **B.** Western blot of fractions immunostained for markers of microsomes (P450 red), mitochondria (Catalase), and perilipin 2 (ADRP). The blot was cut in halves; each half was incubated with different sets of antibodies as indicated. The conditions were as in Fig. S4. Asterisks denote the non-specific bands that originate from incubation with RDH10 antibodies.

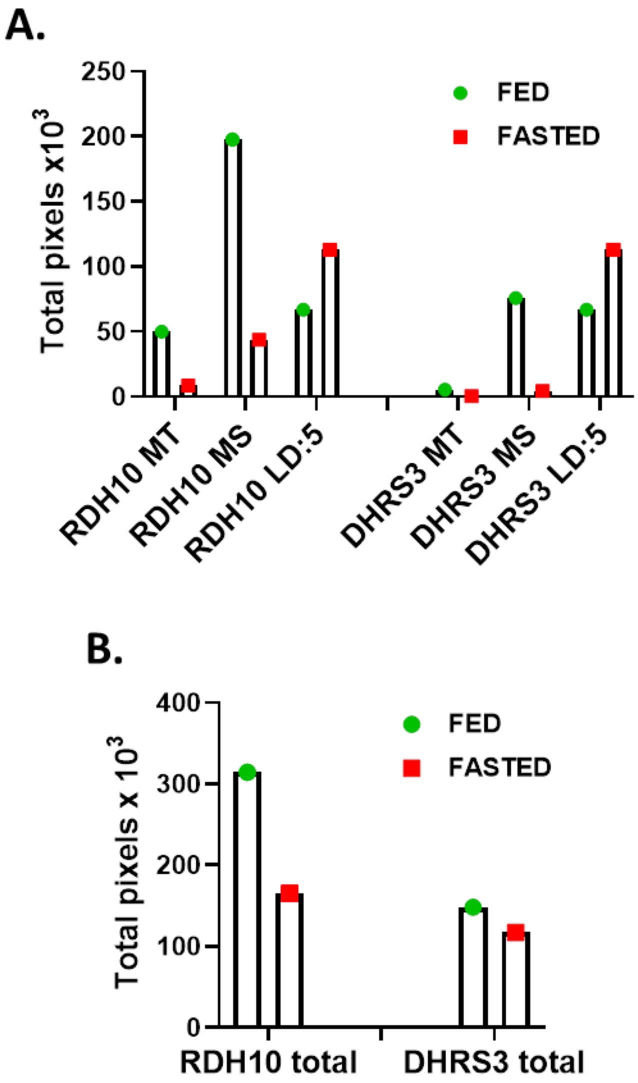

Figure S7. Relative protein levels of RDH10 and DHRS3 in hepatic subcellular fractions of fed *versus* 24 h-fasted mice. A. RDH10 and DHRS3 content in each fraction. The fractions were separated by SDS-PAGE and transferred to nitrocellulose membranes. The blots were aligned and exposed to the same film. The relative intensity of bands was calculated using ImageQuant software. Since the microsomal and mitochondrial fractions were diluted 5-fold, the number of pixels corresponding to lipid droplet fraction was divided by 5 to account for the dilution factor. **B.** The differences in total content of RDH10 and DHRS3 in fed *versus* fasted livers.

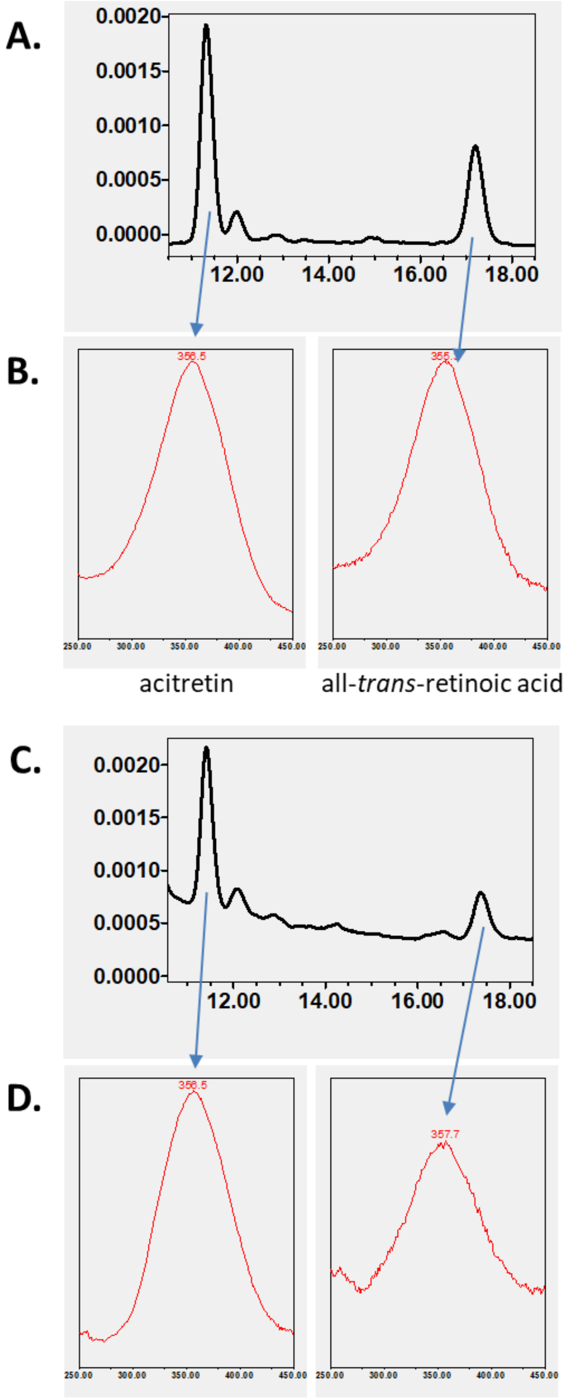

Figure S8. Detection of all-*trans*-retinoic acid in liver samples. HPLC chromatograms show the retention time (**A**) and spectra (**B**) of pure acitretin and pure all-*trans*-retinoic acid and the corresponding characteristics of peaks extracted from mouse liver samples (**C** and **D**). Acitretin was added to liver samples as an internal standard.

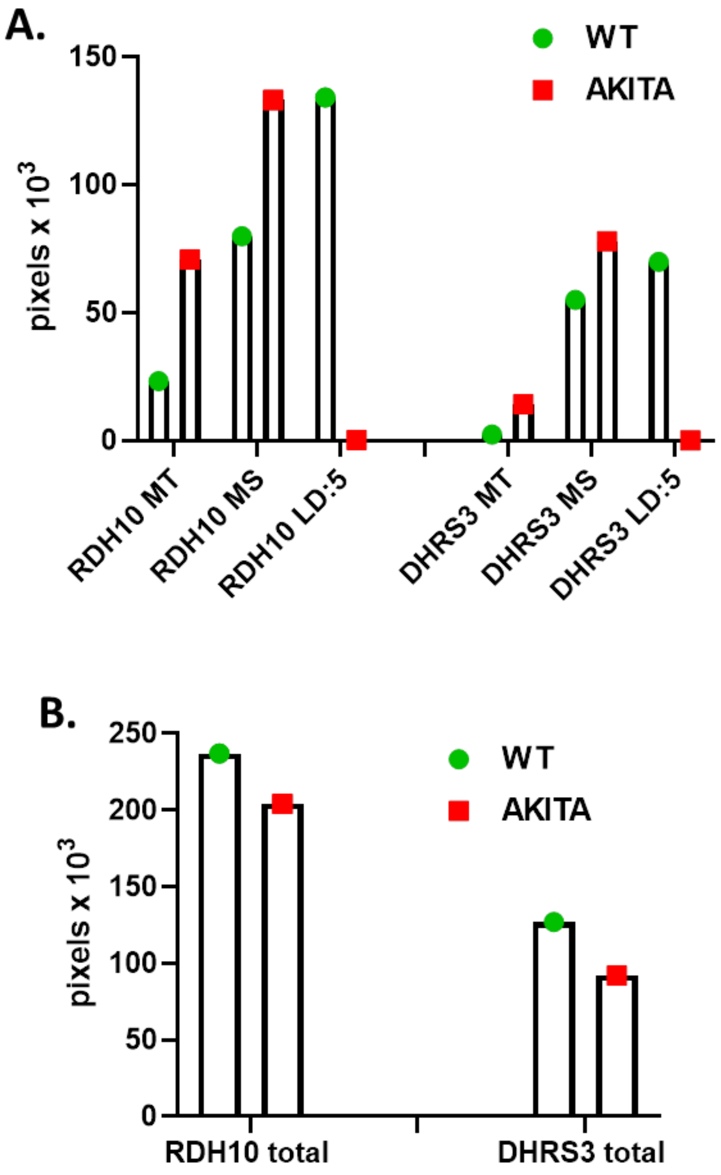

Figure S9. Relative protein levels of RDH10 and DHRS3 in hepatic subcellular fractions of WT *versus* Akita mice. A. Average RDH10 and DHRS3 content in fractions combined from three individual mice. The fractions were separated by SDS-PAGE and transferred to nitrocellulose membranes. The relative intensity of bands was calculated using ImageQuant software. Since the microsomal and mitochondrial fractions were diluted 5-fold, the number of pixels corresponding to lipid droplet fraction was divided by 5 to account for the dilution factor. **B.** The total content of RDH10 and DHRS3 in WT *versus* Akita livers.
